# Evaluation of next generation sequencing platforms for population targeted sequencing studies

**DOI:** 10.1186/gb-2009-10-3-r32

**Published:** 2009-03-27

**Authors:** Olivier Harismendy, Pauline C Ng, Robert L Strausberg, Xiaoyun Wang, Timothy B Stockwell, Karen Y Beeson, Nicholas J Schork, Sarah S Murray, Eric J Topol, Samuel Levy, Kelly A Frazer

**Affiliations:** 1 Scripps Genomic Medicine - Scripps Translational Science Institute - The Scripps Research Institute, N. Torrey Pines Court, La Jolla, CA 92037, USA; 2 The J Craig Venter Institute, Medical Center Drive, Rockville, MD 20850, USA

## Abstract

**Background:**

Next generation sequencing (NGS) platforms are currently being utilized for targeted sequencing of candidate genes or genomic intervals to perform sequence-based association studies. To evaluate these platforms for this application, we analyzed human sequence generated by the Roche 454, Illumina GA, and the ABI SOLiD technologies for the same 260 kb in four individuals.

**Results:**

Local sequence characteristics contribute to systematic variability in sequence coverage (>100-fold difference in per-base coverage), resulting in patterns for each NGS technology that are highly correlated between samples. A comparison of the base calls to 88 kb of overlapping ABI 3730xL Sanger sequence generated for the same samples showed that the NGS platforms all have high sensitivity, identifying >95% of variant sites. At high coverage, depth base calling errors are systematic, resulting from local sequence contexts; as the coverage is lowered additional 'random sampling' errors in base calling occur.

**Conclusions:**

Our study provides important insights into systematic biases and data variability that need to be considered when utilizing NGS platforms for population targeted sequencing studies.

## Abstract 2

Human sequence generated from three next-generation sequencing platforms reveals systematic variability in sequence coverage due to local sequence characteristics.

## Background

The Sanger method [1] of sequencing by capillary electrophoresis using the ABI 3730xL platform has been employed in many historically significant large-scale sequencing projects and is considered the 'gold standard' in terms of both read length and sequencing accuracy [2]. Several next generation sequencing (NGS) technologies have recently emerged, including Roche 454, Illumina GA, and ABI SOLiD, which are able to generate three to four orders of magnitude more sequence and are considerably less expensive than the Sanger method on the ABI 3730xL platform (hereafter referred to as ABI Sanger) [2-4]. To date these new technologies have been successfully applied toward ChIP-sequencing to identify binding sites of DNA-associated proteins [5,6], RNA-sequencing to profile the mammalian transcriptome [7,8], as well as whole human genome sequencing [9-11]. Currently there is much interest in applying NGS platforms for targeted sequencing of specific candidate genes, intervals identified through single nucleotide polymorphism (SNP)-based association studies, or the entire human exome [12-15] in large numbers of individuals.

**Figure 1:**
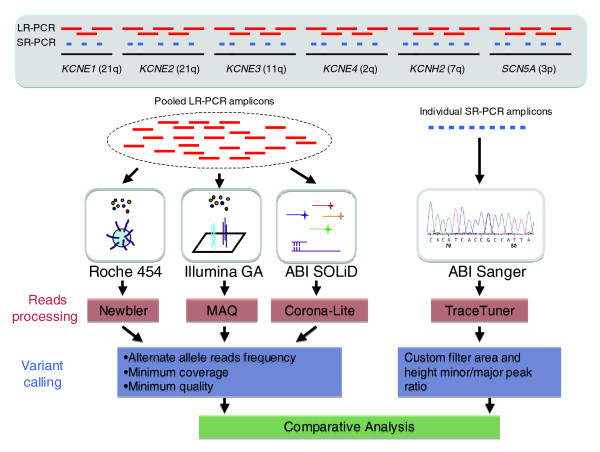
Overview of experimental design. Six genomic intervals, each encoding genes for K^+^/Na^+ ^voltage-gated channel proteins, were amplified using DNA from four individuals and LR-PCR reactions to generate 260 kb of target sequence per sample. Amplicons from each individual were pooled in equimolar amounts and then sequenced using the three NGS platforms. The 260 kb examined in this study is representative of human sequences containing 38% repeats and 4% coding sequence compared with 47% and 1%, respectively, genome-wide. For each sample 88 kb was amplified using short range PCR (SR-PCR) reactions targeting the exons and evolutionarily conserved intronic regions. Each SR-PCR amplicon was individually sequenced in the forward and reverse directions using the ABI-3730xL platform (Additional data file 2). Data generated from the NGS platforms were analyzed to identify bases variants from the reference sequence (build 36) and the quality of the variant calls was assessed using platform specific methodologies. A comparative analysis of the sequence data from the NGS platforms and ABI Sanger was then performed to determine accuracy, and false positive and false negative rates.

As population targeted sequencing studies are initiated, it is important to determine the issues that will be encountered in generating and analyzing data produced by NGS platforms for this application. Here, we generate 260 kb of targeted sequence in four samples using the manufacturer recommended and/or supplied sample library preparation methods, sequence generation, alignment tools, and base calling algorithms for the Roche 454, Illumina GA, and ABI SOLiD platforms (Figure 1). For each NGS technology we generated a saturating level of redundant sequence coverage, meaning that increased coverage is likely to have minimal, if any, effect on data quality and variant calling accuracies. We analyzed the sequences produced by each platform for per-base sequence coverage and for systematic biases giving rise to low coverage. We show that each NGS platform generates its own unique pattern of biased sequence coverage that is consistent between samples. For the short-read platforms, low coverage intervals tend to be in AT-rich repetitive sequences. We also performed a comparative analysis with sequence generated by the well-established ABI Sanger platform (Figure 1) to determine base calling accuracies and how average fold sequence coverage impacts base calling errors. Although the three NGS technologies correctly identify >95% of variant alleles, the average sequence coverage required to achieve this performance is greater than the targeted levels of most current studies.

## Results

### Generation and alignment of sequence reads to targeted intervals

The targeted sequence was amplified in the four DNA samples using long-range PCR (LR-PCR) reactions that were combined in equimolar amounts and sequenced using the three NGS technologies (Figure 1). For the Roche 454 platform we obtained an average of 49,000 reads per sample with an average length of 245 bp (Supplemental Table 1 in Additional data file 1), using Illumina GA we generated an average of 5.9 million reads each 36 bases in length per sample, and using ABI SOLiD we obtained an average of 19.7 million reads each 35 bases in length per sample. Thus, the amount of sequence data generated and analyzed was dependent on the NGS platform and the fraction of the run that was utilized.

The NGS technologies generate a large amount of sequence but, for the platforms that produce short-sequence reads, greater than half of this sequence is not usable. On average, 55% of the Illumina GA reads pass quality filters, of which approximately 77% align to the reference sequence (Supplemental Table 1 in Additional data file 1; Additional data file 2). For ABI SOLiD, approximately 35% of the reads pass quality filters, and subsequently 96% of the filtered reads align to the reference sequence. Thus, only 43% and 34% of the Illumina GA and ABI SOLiD raw reads, respectively, are usable. In contrast to the platforms generating short-read lengths, approximately 95% of the Roche 454 reads uniquely align to the target sequence. When designing experiments and calculating the target coverage for a region, one must consider the fraction of alignable sequence.

### Overrepresentation of amplicon end sequences

**Figure 2:**
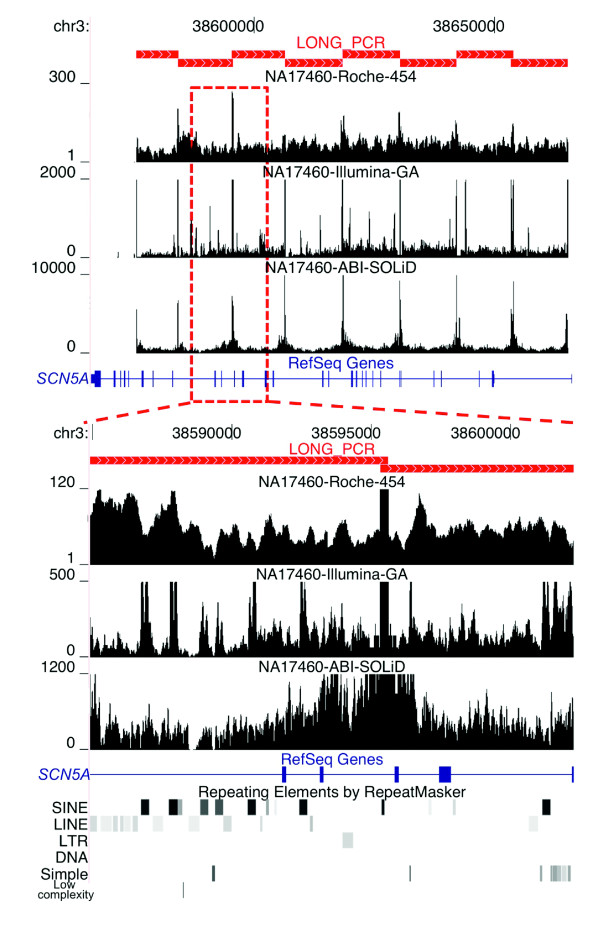
Non-uniform per-base sequence coverage. The 100-kb interval on chromosome 3 encoding the *SCN5A *gene (blue rectangles and joining lines) was amplified using eight LR-PCR amplicons (red filled rectangles in upper panel). On the y-axis, the fold sequence coverage scale is shown for each platform. The upper panel shows that amplicon end sequences are highly overrepresented. The y-axis was set to show the relative fold coverage of the sequences in the interval and therefore does not accurately represent the maximum fold coverage of the amplicon ends, which was 311, 195,473, and 15,041 for Roche 454, Illumina GA, and ABI SOLiD, respectively, in the sample shown. The lower panel shows the non-uniformity of sequence coverage across an approximately 17-kb region encompassing four exons of *SCN5A*. The locations of the repetitive elements (lower black/gray rectangles) in the interval are shown.

In examining the distribution of mapped reads, we observed that the sequences corresponding to the 50 bp at the ends and the overlapping intervals of the amplicons have extremely high coverage (Figure 2; Additional data file 2). These regions, representing about 2.3% (approximately 6 kb) of the targeted intervals, account for up to 56% of the sequenced base pairs for Illumina GA technology. This extreme sequence coverage bias results from overrepresentation of the amplicon ends in the DNA samples after fragmentation prior to library generation. For the ABI SOLiD platform an amplicon end depletion protocol was employed to remove the overrepresented amplicon ends; this was partially successful and resulted in the ends accounting for up to 11% of the sequenced base pairs. For the Roche 454 technology, overrepresentation of amplicon ends versus internal bases is substantially less, with the ends composing only 5% of the total sequenced bases; this is likely due to library preparation process differences between Roche 454 and the short-read length platforms. The overrepresentation of amplicon end sequences is not only wasteful for the sequencing yield but also decreases the expected average coverage depth across the targeted intervals. Therefore, to accurately assess the consequences of sequence coverage on data quality, we removed the 50 bp at the ends of the amplicons from subsequent analyses.

### Sequence coverage of targeted intervals

For each platform we generated a saturating level of redundant sequence coverage, meaning that increased coverage is likely to have minimal, if any, effect on data quality. For the four samples the average sequence coverage depth across the analyzed base pairs is 43×, 188×, and 841× for Roche 454, Illumina GA, and ABI SOLiD, respectively (Supplemental Table 2 in Additional data file 1). For all three NGS technologies there is greater than a hundred-fold variation in the per-base sequence coverage depth (Figure 2). We performed several analyses to determine if the sample preparation method and/or a specific class of sequence elements were responsible for the observed variability (Additional data file 2). We first tested whether the large variability resulted from pooling of the amplicons. For 90% of the amplicons the fold difference in average coverage of unique sequences is less than 2.46, 2.72, and 2.99 on the Roche 454, Illumina GA and ABI SOLiD platforms, respectively (Supplemental Table 3 in Additional data file 1), showing that the error in equimolar pooling or amplicon specific bias (sequence, length) explains only a small fraction of the observed coverage variability. Next we examined how the sequence coverage differs within the individual amplicons. For Roche 454, Illumina GA, and ABI SOLiD the average coefficient of variance was 0.33, 0.9, and 0.73, respectively, for all base pairs, and 0.35, 0.84 and 0.76, respectively, when restricted to unique non-repetitive sequence, defined here as not present in the RepBase database [16]. These results indicate that unique sequences present at equimolar amounts in the library generation step end up being covered at vastly different read depths.

It is important to consider how well the NGS technologies are able to generate sequence reads containing repetitive elements as these sequences comprise approximately 45% of the human genome and may potentially impact genome function. Compared to unique sequences, the Roche 454 technology has a 1.25-fold overrepresentation of LINE elements, Illumina GA has greater than 2-fold higher coverage of SINEs, Alus and simple repeats, while for ABI SOLiD all repetitive elements are covered at approximately half the fold coverage of unique sequences (Supplemental Table 4 in Additional data file 1). Thus, considering all three NGS platforms, Roche 454 generates the most even coverage across both unique and repetitive sequences, Illumina GA shows the most variability in coverage, and ABI SOLiD demonstrates a strong bias against coverage of repetitive elements.

**Figure 3:**
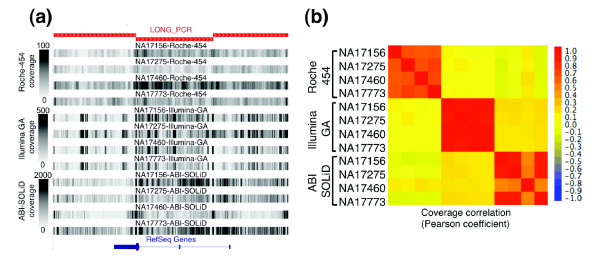
Each NGS technology generates a consistent pattern of non-uniform sequence coverage. **(a) **Sequence coverage depth is displayed as a gray-scale (0-100× for Roche 454; 0-500× for Illumina GA and ABI SOLiD) along an approximately 25-kb region of chromosome 11 amplified by three long-range PCR products (red rectangles). **(b) **A heat-map colored matrix displays the coefficient of correlation of coverage across the entire 260 kb of analyzed sequence between each of the 72 possible pair-wise comparisons (four samples by three technologies). The apparent lower correlation of the Roche-454 sequence coverage is more reflective of the smaller amplitude in the coverage variability (lower average coefficient of variance) than a lack of coverage correlation from sample to sample. The correlation of NA17460 with the other three samples on the ABI SOLiD platform is slightly lower due to technological issues (Additional data file 2) and was therefore excluded from the coefficient of correlation calculation reported in the text.

Interestingly, each NGS technology has a unique reproducible pattern of non-uniform sequence coverage: sequences with high or low coverage in one sample typically had high or low coverage in the other three samples (Figure 3). The coefficient of correlation (*r*) of per-base sequence coverage depth was 0.62, 0.90, and 0.88 between samples on Roche 454, Illumina GA, and ABI SOLiD, respectively. On the other hand, per-base sequence coverage depth for the same sample on different platforms was not well correlated (*r *< 0.19). These data indicate that for all three NGS technologies local sequence characteristics substantially contribute to the observed variability in coverage unique to each technology.

To gain insight into systematic biases of each NGS technology, we examined the sequence composition of intervals with no or low coverage (defined as less than 5% of the average coverage depth; Additional data file 2). Despite having considerably higher average sequence coverage, the ABI SOLiD data have the largest number of no and low coverage intervals (spanning 464 bp and 3,415 bp respectively), the majority of which are AT-rich repetitive sequences (Supplemental Tables 5 and 6 in Additional data file 1). The Illumina GA low coverage regions (spanning 272 bp) also tend to be AT-rich repetitive sequences. Overall, for the short read platforms read depth coverage decreases with increasing AT content, which is consistent with previous studies [17,18] (Supplemental Figure 1 in Additional data file 3). Roche 454 had one no and one low coverage interval (spanning 4 bp and 59 bp, respectively).

### Detection of single nucleotide base variants

We established parameters for calling variant bases in the sequence generated by the NGS technologies based on optimized concordance with the variant calls in the ABI Sanger data. As previously observed, PCR sample preparation can produce imbalanced amplification of the two alleles for some amplicons, resulting in incorrect genotype calls at variant bases by specifically calling heterozygous sites as homozygous sites [19]. Imbalanced amplification is usually suspected to result from polymorphisms in or near the oligonucleotide priming sites that result in greater efficiency of amplification for one of the alleles. To measure this phenomenon in our sample preparation method, we looked at the alternate allele read frequency (AARF; Additional data file 2) at ABI Sanger identified heterozygous positions in the sequence data for the three NGS platforms. Out of the 28 amplicons in this study, four demonstrated allelic imbalances in amplification for one or more samples (Supplemental Table 7 in Additional data file 1). We removed the sequence data for these four amplicons from the variant quality analysis so as to focus on errors caused by the NGS platforms and thereby not have the analysis confounded by sample preparation issues.

### Accuracy of sequence variant calls compared to microarray genotype calls

Accuracy of the variant calls in the NGS and ABI Sanger data for the four samples was initially assessed by comparison to genotype calls for approximately 80 SNPs located in the sequenced intervals and assayed by the Illumina Hap550 BeadChip. The genotype accuracy of the four platforms is 97.4%, 100%, 99.7%, and 98% for Roche 454, Illumina GA, ABI SOLiD and ABI Sanger, respectively (Supplemental Tables 8 and 9 in Additional data file 1). These data show a greater number of discordant genotypes for Roche 454. It is important to note that comparison between sequence and SNPs genotyped on commercial arrays is not expected to be fully indicative of NGS platform variant base calling accuracy in genomic sequences at large. First, false positive rates cannot be considered by SNP microarray technologies because novel variants are not detected. Second, SNP microarrays typically query a subset of 'well behaved' bases; hence, false negative rates based on microarray technology can be underestimated.

### Variant detection comparing NGS to ABI Sanger

**Figure 4:**
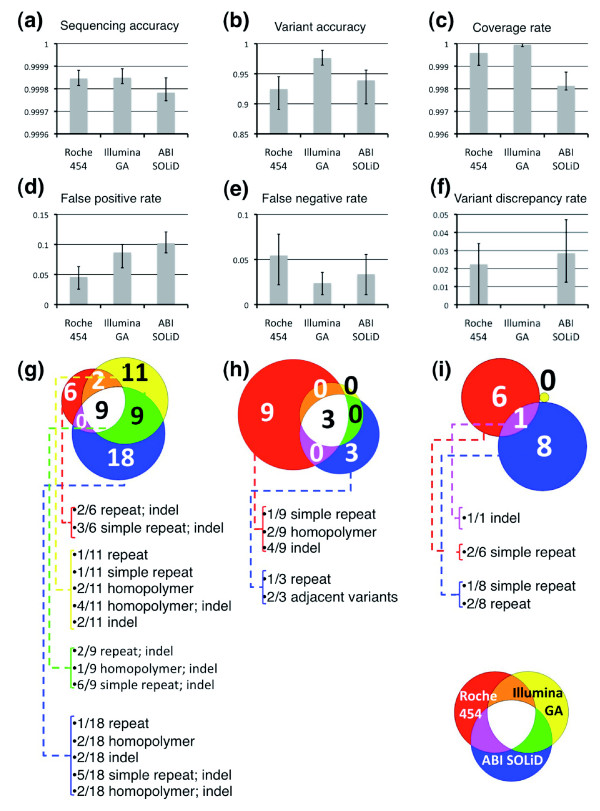
Performance metrics of NGS technologies. **(a-f) **Error bars represent minimum and maximum values obtained from the four samples. **(g-i) **Venn diagram representation of false positive calls (g), false negative calls (h) and discrepant variants calls (i). The inset caption displays the color-coding of each NGS technology and overlaps: for Roche 454 (red), Illumina GA (yellow) and ABI SOLiD (blue). For each NGS platform the number of base calls with errors associated with specific sequence contexts is given (repeat = repetitive element). When two sequence contexts are present they are both listed.

To further assess sequence quality, we next performed a four-way comparison of the base calls generated from the three NGS technologies and ABI Sanger. The identification of heterozygous and homozygous alternate loci was performed in 258,879 base pairs analyzed from all four samples (Supplemental Table 10 in Additional data file 1). There were twenty loci for which the three NGS technologies were concordant in their base calls but discordant with the ABI Sanger calls. Visual inspection of the ABI Sanger traces revealed that eight of these loci represented base calling errors in the original data, thereby resolving the discrepancy. However, for 12 loci (9 false positive and 3 false negative calls) the discrepancies were not resolved (Figure 4g,h). Two of the discrepant calls were assayed by the Illumina Hap550 array (Supplemental Table 9 in Additional data file 1) and their calls were concordant with the NGS platforms. We examined the genotypes of the remaining discrepant calls by independent Sanger sequencing. As previously established [19,20], errors in Sanger sequencing of human diploid DNA are approximately 7% and result from: PCR primers sometimes overlapping unknown DNA variants leading to imbalanced amplification of the two alleles; and difficulty of automated software to correctly call heterozygous sites. Thus, replicating the Sanger sequencing with different PCR and sequencing primers and manual inspection of the traces can be considered an independent measurement. We successfully examined eight of the discrepant calls using this approach, of which seven agreed with the calls made by the NGS platforms (Supplemental Figure 3 in Additional data file 3). In total, nine of the ten discrepant calls investigated (two by genotyping and seven by Sanger sequencing) were confirmed as being incorrect in the original ABI-Sanger sequencing. As a result of this analysis for the first time by comparison with NGS technologies, the ABI Sanger false positive and false negative rates for human diploid DNA are estimated to be approximately 0.9% and approximately 3.1%, respectively. These 12 loci identified as ABI Sanger errors were removed from consideration when assessing the NGS technologies' performance.

We next calculated five different performance metrics (sequencing accuracy, variant accuracy, false positive rate, false negative rate, and variant discrepancy rate) for the NGS platforms (Supplemental Table 11 in Additional data file 1). Sequencing accuracy, which measures the concordance of all calls including homozygous reference, was greater than 99.99% for all NGS technologies (Figure 4a). On the other hand, variant accuracy, which measures the ability of NGS technologies to make a correct call at known variant positions identified by ABI Sanger, was lower, averaging over the four individuals for each technology at 95%, 100%, and 96% for Roche 454, Illumina GA, ABI SOLiD, respectively (Figure 4b). The false positive rate of Roche 454, Illumina GA and ABI SOLiD is approximately 2.5%, approximately 6.3%, and approximately 7.8%, respectively; the false negative rates are approximately 3.1%, approximately 0%, and 0.9% (Figure 4d,e). We also examined the variant discrepancy rates, which reflect the number of positions that have been correctly identified as variant, but assigned incorrect zygosity. For Roche 454, Illumina GA, and ABI SOLiD the variant discrepancy rates were 2%, 0%, and 3%, respectively. These five performance metrics indicate that at saturating sequence coverage and the methodologies employed to call variants, the short-read platforms have greater sensitivity but lower specificity than Roche 454.

In examining the sequences underlying false positive and false negative calls in the NGS technologies, we determined that these errors were unexpectedly not associated with low sequence coverage but rather are the result of systematic biases (Figure 4g,h,i). For each NGS platform, 47% of the bases with an error in one sample had an error in at least one other sample (Supplemental Table 12 in Additional data file 1). Greater than 72% of these false positive and negative calls are associated with at least one and >33% with two of the following sequence contexts: repetitive elements; a homopolymer stretch ≥6 bases; simple repeats; the presence of an indel within 30 bp. These sequence contexts likely present significant challenges during read alignment, especially for the short-read technologies, resulting in variant detection errors. Two out of the three false negatives specific for the ABI SOLiD platform were due to the inability to detect adjacent SNPs with existing variant calling software applied to color-space sequencing technology (Additional data file 2).

### Detection of indels

Detection of heterozygous indels remains a technological challenge using the ABI Sanger platform [21]. Here the ABI Sanger sequencing detected 11 heterozygous indels in the 88 kb of sequence analyzed. The Roche 454 technology successfully identified five of these indels, all of which ranged from 3-16 bp in length (Supplemental Table 13 in Additional data file 1). Of the six indels missed by Roche 454, five were single base in length in homopolymer sequences, and one was a 15 bp insertion that was not completely resolved due to low coverage. Interestingly, Roche 454 identified 43 additional indels in the 88 kb of overlapping ABI Sanger sequences (Supplemental Table 14 in Additional data file 1). Bearing in mind that the false positive rate for these data cannot be estimated, this suggests that the Roche 454 platform may be more useful for identifying indels than the ABI Sanger technology. The Illumina GA and ABI SOLiD platforms at the time of this analysis were unable to identify indels automatically.

### Assessing performance metrics at lower coverage

**Figure 5:**
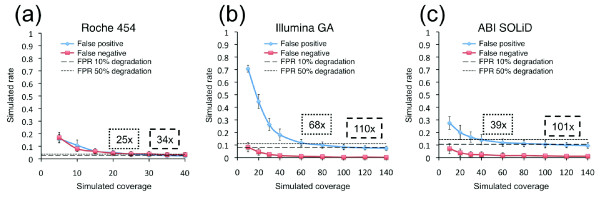
False positive rates (FPRs) and false negative rates for the three NGS technologies at simulated varying coverage depths. Performances of **(a) **Roche 454, **(b) **Illumina GA, and **(c) **ABI SOLiD at lower coverage depths were simulated by random subsampling of the reads. Error bars represent the standard deviation over the four samples for ten iterations. The thresholds for a 10% and 50% error rate degradation of the minimum false positive rate are indicated by dashed and dotted lines, respectively, and the corresponding coverage depth reported in dashed and dotted boxes, respectively.

To efficiently perform population-based targeted sequencing studies using NGS technologies, it is important to determine the lowest average sequence coverage required to achieve a specified sensitivity and specificity. To estimate this coverage requirement, we simulated varying coverage depths for all three technologies, recalled genotypes, and calculated false positive and false negative rates for each coverage depth (Additional data file 2). The maximum simulated average coverage was 40-fold for Roche 454 and 140-fold for both Illumina GA and ABI SOLiD. The false positive error rates are more impacted by low coverage compared with false negative rates; thus, we focused our analysis on the former. The average coverage depth for 50% false positive error rate degradation (percentage of the minimum simulated error rate; see Materials and methods) is achieved at 25-fold, 68-fold, and 39-fold and for 10% degradation at 34-fold, 110-fold and 101-fold for Roche 454, Illumina GA, and ABI SOLiD, respectively (Figure 5). These results indicate that the short-read technologies have a two- to three-fold greater sequence coverage depth requirement relative to Roche 454. Thus, errors at high coverage are systematic and typically associated with specific sequence contexts; at lower coverage errors result from random sampling in base calling. Consistent with this observation, the performance of the NGS technologies at low sequence coverage is correlated with per-base sequence coverage uniformity; the Illumina GA, which has the highest coverage variability, performs the worst at lower coverage, whereas Roche 454, with the most uniform coverage, performs the best. This observation suggests that for all the NGS technologies, achieving more uniform sequence coverage would result in considerably higher performance at lower coverage.

## Discussion

Our study highlights many issues encountered as NGS platforms are utilized for population-based targeted sequencing studies, including biases in sample library generation, difficulties mapping short reads, variation in sequence coverage depth of unique and repetitive elements, difficulties detecting indels with short reads, the systematic errors of the NGS technologies and the impact of all these features on variant calling accuracy. We note that the results of our analyses reported for each NGS platform are the combined effects of the manufacturer recommended laboratory methods, sequence read alignment tools, and base calling algorithms utilized.

At high sequence coverage all NGS platforms have excellent variant calling accuracy (>95%) as assessed by the detection of known SNP variants. However, this accuracy is lower than the values typically stated for the NGS platforms [22-25]. NGS-reported accuracies are typically being measured, in human sequences, by comparison to commercial SNP genotyping arrays, which we demonstrate are inadequate for ascertaining false positive and false negative rates. Therefore, the sequence-based accuracies reported here are likely to be more indicative of the real performance of NGS platforms for *de novo *detection of variants in human sequences.

Interestingly, our analysis indicates that ABI Sanger has a false negative rate of approximately 3%, which is comparable to the three NGS technologies at saturating coverage. Thus, there are likely many more DNA polymorphisms yet to be detected in human samples [26]. Indeed, heterozygous indel detection, which is difficult using PCR-based sample preparation methods and ABI Sanger sequencing [27], may be easier to achieve using NGS platforms because each allele is sequenced and detected independently. This is especially important since indel variants constitute approximately 25% of the reported mutations implicated in human disease [28] and their identification would precede a more complete understanding of how they determine human phenotypes.

The saturating sequencing coverage we exploited enabled the determination of the sequence coverage threshold below which false discovery rates of variants were unacceptably high. This revealed that for accurate detection of biallelic sites, the average depth of sequence coverage required for all three NGS platforms but especially for the short-read technologies is considerably higher than the empirically determined coverage of 20-fold utilizing random Sanger sequencing [29]. This coverage requirement for NGS technologies is further supported by a recent multiplexed targeted resequencing study that showed that accurate detection of variant loci necessitates a 20-fold read depth per base, and a higher average depth due to coverage variability [30], and a recent yeast mutational profiling study that showed 10-15-fold coverage is required to detect variants in haploid organisms [31]. Importantly, these required average sequence coverages are much higher than what is typically employed in targeted sequencing studies utilizing NGS technologies.

## Conclusions

Our results suggest that to effectively balance cost and data quality for population targeted sequencing studies, there are two key aspects of NGS technologies that need optimization: the uniformity of per-base sequence coverage must be improved to reduce the total amount of sequence generation required; and the systematic errors that impact variant calling accuracy need to be reduced so that the false positive and false negative rates are acceptable for sequence-based association studies. Although recent improvements in the NGS platforms, such as paired end and longer reads, will mitigate these issues, all aspects of the NGS platforms, laboratory methods, sequence alignment tools, and base calling algorithms partially contribute to the problems and, therefore, need to be simultaneously optimized.

## Materials and methods

### Sample preparation

Twenty-eight LR-PCR reactions were performed to amplify six genomic intervals spanning a total of 266 kb in each of four DNA samples (NA17275, NA17460, NA17156, and NA17773) obtained from the Coriell Institute [32] (Additional data file 2). Following LR-PCR, the 28 amplicons generated using a single DNA sample template, ranging in size from 3,088 bp to 14,477 bp, were quantified, combined in equimolar amounts, and used to create libraries for Roche 454, Illumina GA and ABI SOLiD sequencing.

### Roche 454

The Roche 454 laboratory methods and protocols used were as described by Rothberg and coworkers [23]. The reads produced by the Roche 454 FLX platform were mapped to the reference sequence using the algorithm Newbler version 1.1.03.19 (provided by Roche), unless stated otherwise.

### Illumina GA

The Illumina GA libraries were prepared according to the manufacturer's instructions from the 28 equimolar pooled PCR products except for the fragmentation step (Additional data file 2). The Illumina GA reads were aligned with MAQ 0.6.2 [33], unless stated otherwise.

### ABI SOLiD

Long mate pair (LMP) libraries DNA libraries were generated from the four 28 equimolar pooled amplicon samples and end sequenced using standard ABI SOLiD protocols at Applied Biosystems in Beverly, MA. For each sample, ABI aligned the sequence reads to the reference sequence and mate-pairing information was not employed in this project. The aligned reads and the number of calls per base for each position were used for data analysis (Additional data file 2).

The LMP library construction process requires more DNA amplification and manipulation and is useful for the detection of indels and structural variants. Therefore, as opposed to the library construction processes for Roche-454 and Illumina GA, which were focused on read fragment preparation alone, discarding mate-pair information from the LMP protocol reads and using them as unpaired reads may have introduced mapping biases when used to detect SNPs. Indeed, the generation of these libraries creates variable tag lengths that require different mapping techniques to ensure proper representation of the genome. Shorter tags will not map with a 35 bp and 3 mismatches schema and as a result substantial portions of the genome can be differentially sampled due to fixed mapping criteria.

These differences in the library techniques emphasize the need for the use of quality score information in the ABI SOLiD reads to properly trim the data before mapping and allow for proper comparison to a Roche 454 and Illumina GA data that currently perform Keypass, Chastity and Purity filtering of the data before SNP calling.

### Calling genotypes in the NGS sequence data

We define the alternate allele as the most commonly called base (which is not the reference base) for a given position in the reference sequence. Then, the AARF is the fraction of reads corresponding to the alternate allele.

Positions called as reference homozygote by ABI Sanger have AARFs close to 0% by the NGS technologies (Supplemental Figure 2 in Additional data file 3). Also, positions called as alternate homozygous by ABI Sanger have AARFs near or at 100% by the NGS technologies. The AARFs for heterozygous calls by ABI Sanger is centered at 50% for Roche 454 and Illumina GA; for ABI SOLiD it is centered at 42% (Additional data file 2). Upon independent inspection of the three technologies, most ABI Sanger-called heterozygotes fell in the range 20-80%. Thus, for the NGS technologies, utilizing only high quality bases we call positions with AARFs between 20% and 80% as heterozygous, positions with AARFs >80% as homozygous alternate, and positions with AARFs <20% as homozygous reference (Additional data file 2).

### Short-range PCR and Sanger sequencing

We used an existing data set deposited by JCVI and performed under the auspices of the National Heart, Lung and Blood Re-sequencing and Genotyping program [34]. The data set included 88 kb of non-contiguous sequence encompassing the exons and the intronic sequence conserved with mouse and rat in the K^+^/Na^+ ^channel proteins produced by employing 273 short-range PCR reactions generating amplicons averaging 418 bp in length.

### Definitions of performance metrics

In order to assess the performance of the sequencing technologies, we define several metrics.

#### Comparing a genotyping microarray to a sequencing technology

##### Genotype accuracy

We genotyped the four samples on the Illumina Hap550 microarray according to specifications of the manufacturer. We compared the genotype calls of the SNPs on the Hap550 microarray with the genotypes observed from sequencing (Supplemental Table 8 in Additional data file 1). Genotype accuracy is defined as: (Number of genotypes matching exactly between Illumina Hap550 and a sequencing technology)/(Number of compared positions).

#### Metrics for comparing a NGS sequencing technology with ABI Sanger

We initially assumed the ABI Sanger sequence data are correct because it is an established method with the longest history [2]. Upon further analysis, we found that this assumption was not always true; there were some positions incorrectly called by ABI Sanger, but correctly called by the NGS technologies (see Results). We refer to Table 1 annotations to clarify these definitions.

**Table 1:** Annotations of the genotypes differences to illustrate the definition of the metrics used to compare ABI Sanger and NGS Technologies

	Sanger			
	
NGS technology	Homozygous reference	Heterozygous	Homozygous alternate	N/N
Homozygous reference	A1	A2	A3	A4
Heterozygous	B1	B2	B3	B4
Homozygous alternate	C1	C2	C3	C4
N/N	D1	D2	D3	D4

N/N: positions at which genotype was not called.

##### Sequencing accuracy

This is defined as the number of concordant calls between ABI Sanger and a NGS technology. Following the diagram above, this is calculated as (A1 + B2 + C3)/Total, where Total is defined as the number of positions with genotype calls by both technologies, or (A1 + A2 + A3 + B1 + B2 + B3 + C1 + C2 + C3). Because the sequencing accuracy metric is dominated by the concordance of a large number of homozygous reference calls (A1), this metric tends to be very near 1.

##### Variant accuracy

Because 'sequencing accuracy' tends to be dominated by the large number of homozygous reference calls, we define another metric called 'variant accuracy'. Variant accuracy is restricted to the variant positions called by ABI Sanger and is defined as: (B2 + C3)/(A2 +A3 + B2 + B3 + C2 + C3).

##### False positive rate of variants (false positive rate)

We define a false positive when the NGS technology calls a variant where ABI Sanger calls a homozygous reference. The false positive rate is calculated as (B1 + C1)/(B1 + B2 + B3 + C1 + C2+ C3).

##### False negative rate of variants (false negative rate)

We define a false negative when ABI Sanger detects a variant, but the NGS method calls this locus as a homozygous reference. The false negative rate is calculated as (A2 + A3)/(A2 + A3 + B2 + B3 + C2 + C3).

##### Variant discrepancy rate

We define the variant discrepancy rate as (B3 + C2)/(B2 + B3 + C2 + C3). This metric reflects ABI Sanger variant positions that are also detected by the NGS technology, but where the genotype calls disagree.

##### Coverage rate

The fraction of positions with genotype calls is defined as 1-(D1 + D2 + D3)/(A1 + A2 + A3 + B1 + B2 + B3 + C1 + C2 + C3 + D1 + D2 + D3).

##### ABI Sanger false positive rate

We define a ABI Sanger false positive when ABI Sanger calls a variant but all three NGS technologies call the locus as homozygous reference. We assume the NGS technologies to be correct, and this was confirmed by re-inspection of the ABI Sanger traces. The ABI Sanger false positive rate is calculated as follows. The numerator is the number of loci that are called as homozygous reference by all three NGS technologies, but as a variant in ABI Sanger. In the denominator, we consider all positions that were called as variant by Sanger and also had a genotype call by all three NGS technologies.

##### ABI Sanger false negative rate

We define a ABI Sanger false negative as a locus where the initial call by ABI Sanger is homozygous reference but all three NGS technologies detect a variant at this locus. In the numerator of the ABI Sanger false negative rate, we count the number of variant loci that are identified by all three NGS technologies but called as homozygous reference by ABI Sanger. We note that zygosity may not agree among the three NGS technologies, but if all three technologies identify a variant at the position, the locus is included (Supplemental Table 12 in Additional data file 1). The denominator represents the number of loci called as variant by all three NGS technologies (although the zygosity may differ).

### Validation of genotypes discordant between ABI Sanger and the three NGS platforms

PCR reactions were performed in 50 μl platinum buffer (Invitrogen, Carlsbad, CA, USA) using 5 pM of primers (Supplemental Table 15 in Additional data file 1) and 0.2 μl of platinum *Taq *DNA polymerase, incubated 2 minutes at 94°C followed by 35 cycles at 30 s at 94°C, 30 s at 60°C and 30 s at 72°C, followed by 5 minutes at 72°C for final elongation. PCR products were purified using QIAquick PCR purification columns (Qiagen, Hilden, Germany)) and sequenced in both directions using the same primers as in the PCR and Big Dye terminator sequencing chemistry (Applied Biosystems, Foster City, CA USA).

### Simulations

Simulations were performed in order to assess performance of each NGS platform at lower coverage depths. For each simulation, we randomly sampled a subset of the reads and recalled genotypes. The size of the subset was determined by the desired coverage depth.

### Inferring coverage at various error rate degradations

To obtain the coverage depths in Figure 5, we first examined the error rate at the maximal simulated coverage. For 50% error rate degradation, we multiplied the error rate at the maximal coverage by 1.5 to get the desired error rate. For 10% error rate degradation, we multiplied the error rate at the maximal coverage by 1.1 to get the desired error rate. We then examined the error rates from the simulations at different coverage depths, and interpolated what coverage depth corresponds to the desired error rate. For example, the false positive error rate for Illumina GA at 140× from the simulations is 0.073. At 50% error rate degradation, the false positive rate is 0.110. The false positive rates at coverage depths of 60× and 80× are 0.118 and 0.099, respectively, so we know that a coverage depth within the range of 60× to 80× will give a false positive rate of 0.110. Using linear interpolation, we deduce that a coverage depth of 68× gives a false positive rate of 0.110, and this is reported in Figure 5.

The error rates for Illumina GA and ABI SOLiD at maximum simulated coverage are slightly higher than what was experimentally observed. The additional errors observed in the simulations are largely associated with low coverage regions and are different between iterations, whereas the systematic errors present in the experimental data set at full coverage are shared between iterations. This shows that the simulation produces random sampling errors, directly associated with low coverage regions.

## Abbreviations

AARF: alternate allele read frequency; LR-PCR: long-range PCR; NGS: next generation sequencing; SNP: single nucleotide polymorphism.

## Competing interests

PCN and SS Murray currently hold stock in Illumina, Inc.

## Authors' contributions

OH, PN, and SL performed next gen sequencing analysis; SL, and TS performed Sanger sequencing analysis; XW, and KB performed next gen sequencing experiments; KF, ET, NS, RS, SL, and SM facilitated the study; KF, SL, PN, and OH designed the study and wrote the manuscript.

## Additional data files

The following additional data are available with the online version of this paper: a PDF including 15 supplemental tables (Additional data file 1); supplemental Materials and methods (Additional data file 2); a PDF including three supplemental figures (Additional data file 3).

## Supplementary Material

Additional data file 1 Supplemental Table 1: result of the NGS pipeline analysis. Supplemental Table 2: coverage information of all the samples in each NGS. Supplemental Table 3: coverage variability between amplicons. Supplemental Table 4: coverage variability between different repeat elements. Supplemental Table 5: amount of sequence with no or low coverage. Supplemental Table 6: list of regions with no or low coverage for each NGS. Supplemental Table 7: analysis of the allelic imbalance amplification of the LR-PCR amplicons. Supplemental Table 8: result of the comparison between sequencing and genotyping on Illumina Hap550. Supplemental Table 9: loci with discrepant calls between genotyping and sequencing. Supplemental Table 10: genotype calls comparison between ABI Sanger and each NGS. Supplemental Table 11: quality metrics of the comparison of the genotype calls between ABI Sanger and NGS technologies. Supplemental Table 12: all the loci with discordant genotype calls between ABI Sanger and NGS Technologies. Supplemental Table 13: all indel loci identified by ABI Sanger and their calls in Roche 454. Supplemental Table 14: all indels identified by Roche 454 and missed by ABI Sanger. Supplemental Table 15: all the primers and oligonucleotides used in the study. Click here for file

Additional data file 2 Additional information to the methods used and calculations performed for this study. Click here for file

Additional data file 3 Supplemental Figure 1: coverage distribution as a function of GC content for the 3 NGS. Supplemental Figure 2: distribution of alternate allele read frequency obtained for each NGS. Supplemental Figure 3: Sanger Traces of eight loci found to have discrepant calls between Sanger and NGS. Click here for file
