# Supplementary material for: Evaluation of next generation sequencing platforms for population targeted sequencing studies
Source: Genome Biol. 2009 Mar 27;10(3):R32. doi: 10.1186/gb-2009-10-3-r32 (PMC2691003; doi:10.1186/gb-2009-10-3-r32)
Supplement: Additional data file 1 — Supplemental Table 1: result of the NGS pipeline analysis. Supplemental Table 2: coverage information of all the samples in each NGS. Supplemental Table 3: coverage variability between amplicons. Supplemental Table 4: coverage variability between different repeat elements. Supplemental Table 5: amount of sequence with no or low coverage. Supplemental Table 6: list of regions with no or low coverage for each NGS. Supplemental Table 7: analysis of the allelic imbalance amplification of the LR-PCR amplicons. Supplemental Table 8: result of the comparison between sequencing and genotyping on Illumina Hap550. Supplemental Table 9: loci with discrepant calls between genotyping and sequencing. Supplemental Table 10: genotype calls comparison between ABI Sanger and each NGS. Supplemental Table 11: quality metrics of the comparison of the genotype calls between ABI Sanger and NGS technologies. Supplemental Table 12: all the loci with discordant genotype calls between ABI Sanger and NGS Technologies. Supplemental Table 13: all indel loci identified by ABI Sanger and their calls in Roche 454. Supplemental Table 14: all indels identified by Roche 454 and missed by ABI Sanger. Supplemental Table 15: all the primers and oligonucleotides used in the study. [file gb-2009-10-3-r32-S1.pdf]

Supplemental Table 1. Summary of Roche 454, Illumina GA, and ABI SOLiD sequence reads. Raw reads were generated, filtered for quality, and then aligned to the reference sequence (Supplemental Methods). All subsequent analyses in this study are based on using the Newbler algorithm to align Roche 454 reads and the MAQ algorithm to align Illumina GA reads.

		NA17156		NA17275		NA17460		NA17773	
Roche 454	No. raw sequence reads	47,968		31,669		71,580		46,244	
	Alignment tool	Newbler	BLAST	Newbler	BLAST	Newbler	BLAST	Newbler	BLAST
	Aligned reads (% of raw)	95.6	83.7	94.7	80.6	95.6	82.2	95.9	82.1
Illumina GA	No. raw sequence reads	6,682,085		5,979,236		5,426,181		5,315,215	
	Reads passing filters (% of raw)	52.26		53.16		57.18		58.36	
	Alignment tool	ELAND	MAQ	ELAND	MAQ	ELAND	MAQ	ELAND	MAQ
	Aligned reads (% of filtered)	84.61	78.72	88.42	82.36	80.95	77.89	71.17	70.16
ABI SOLiD	No. raw sequence reads	20,426,667		15,318,798		16,023,158		26,846,896	
	Reads passing filters (% of raw)	40.39		35.29		28.84		35.63	
	Aligned reads (% of filtered)	96.71		96.51		95.84		96.63	

Supplemental Table 2. Sequence coverage of targeted intervals in the four samples (Supplemental Methods).

		NA17156	NA17275	NA17460	NA17773
	Targeted bp	266,201			
	Sequenced bp	251,973	255,057	266,201	266,201
	Analyzed bp	245,892	248,980	260,036	260,036
Roche 454	≥10x	242,857 (98.77)	243,085 (97.63)	258,450 (99.39)	257,623 (99.07)
	0x	0	0	0	61
	Avg. coverage	43	28	61	39
Illumina GA	≥10x	245,778 (99.95)	248,960 (99.99)	259,626 (99.84)	258,578 (99.82)
	0x	0	0	0	5
	Avg. coverage	203	263	157	130
ABI SOLiD	≥10x	245,197 (97.2)	248,112 (99.7)	259,427 (99.8)	259,272 (99.7)
	0x	88	153	138	85
	Avg. coverage	1036	671	537	1121

Supplemental Table 3. Sequence coverage variability between amplicons. The fold difference and distribution in sequence coverage between amplicons for each NGS technology (Supplemental Methods).

		Max	95th percentile	5th percentile	Min	95th to 5th percentile ratio
Roche 454	NA17156	1.68	1.37	0.63	0.28	2.16
	NA17275	1.43	1.35	0.61	0.33	2.22
	NA17460	2.11	1.52	0.62	0.48	2.46
	NA17773	1.54	1.36	0.72	0.58	1.90
Illumina GA	NA17156	1.55	1.48	0.63	0.42	2.34
	NA17275	1.42	1.34	0.49	0.43	2.74
	NA17460	1.61	1.56	0.60	0.31	2.62
	NA17773	1.53	1.24	0.57	0.34	2.17
ABI SOLiD	NA17156	1.47	1.43	0.48	0.37	2.99
	NA17275	1.41	1.31	0.75	0.73	1.74
	NA17460	3.16	1.53	0.78	0.56	1.97
	NA17773	1.89	1.64	0.60	0.40	2.75

Supplemental Table 4. Sequence coverage of repetitive elements. Relative coverage of repetitive to unique sequences across the 260-kb analyzed interval. Standard deviation corresponds to the 4 samples variation.

	bp per sample	Roche 454	Illumina GA	ABI SOLiD
All repeats	101851	1.13+/-0.03	1.23+/-0.09	0.68+/-0.06
LINE	47030	1.25+/-0.06	0.78+/-0.03	0.56+/-0.03
SINE	33214	1.04+/-0.04	2.08+/-0.21	0.70+/-0.03
Alu	22263	1.02+/-0.06	2.63+/-0.3	0.56+/-0.03
Simple repeats	2462	0.99+/-0.12	2.19+/-0.18	0.65+/-0.08
Low Complexity repeats	1051	0.89+/-0.09	0.84+/-0.08	0.48+/-0.14

Supplemental Table 5A. No Coverage Regions. Regions are counted multiple times if they have 0 coverage in more than one individual.

	No. of Regions	Total Number of base pairs with no coverage	Fraction of Regions that are Repetitive	% AT, averaged across regions
Roche 454	1[a]	59	0/1	29%
Illumina GA	2	5	2/2	58%
ABI SOLiD	40	464	30/40	90%

Supplemental Table 5B. Low Coverage Regions. For details see Supplemental Table 6.

	No. of Regions	Total Length of Low Coverage Regions (bp)	Fraction of Regions that are Repetitive	% AT, averaged across regions
Roche 454	1	4	0/1	25%
Illumina GA	13	272	11/13	82%
ABI SOLiD	168	3415	128/168	85%

[a]Raw 454 reads with low-quality bases aligned to the 59 bp interval, but no high-quality reads aligned to the this interval (see Supplemental Methods).

Supplemental Table 6. Detailed analysis of low and no coverage regions

NGS	coverage type	NCBI36 coordinates	coverage type details	Sequence	repeats	%AT	length
454	low coverage	chr7:150286181-150286184	≤2reads in ≥2 samples	GAGC		25	4
	no coverage	chr7:150298287-150298345	no coverage in NA17773	CACACACCCCCAAGCTGGCCCTTCTGCTGCCCCATGGCCCCCCTCCCCAGAGCCCAAGG		29	59
Illumina GA	low coverage	chr3:38588463-38588538	≤9 reads in ≥2 samples	TAATATATAAATATGCACTTATATAGGTGCATATTATATATTATAATATATACTTATATTTATATATATAGGTTTATA	LINE	87	76
		chr21:34764577-34764636		GTAAAATAATAATTACTGAAACTGATAAACAATATTAATAATTTTAATAGCCATTAAATT		85	60
		chr7:150286423-150286446		CGGGGCGCCCGCGCCGCCCGCGCC	Low complexity	0	24
		chr3:38588597-38588620		TAAAGATATATTCATATGTGAATA	LINE	83	24
		chr21:34769880-34769903		TGAAAAAAATTTATAAATTATACT	LINE	92	24
		chr3:38588657-38588673		AATATATTTTTTATATT	LINE	100	17
		chr3:38585279-38585295		TATCATTTTATATGATT	LINE	88	17
		chr21:34752251-34752258		TTTATATT	Low complexity, Simple repeat	100	8
		chr7:150286125-150286130		TGGCCT		33	6
		chr3:38589382-38589385		TATA	Simple repeat	100	4
		chr3:38589328-38589331		ATAT	Simple repeat	100	4
		chr11:73842475-73842478		TATA	Simple repeat	100	4
		chr11:73839652-73839655		TAAT	Low complexity	100	4
	no coverage	chr3:38588492-38588494	no coverage in NA17773	CAT	LINE	66	3
		chr21:34764600-34764601		GA	Low Complexity	50	2
SOLiD	no coverage	chr11:73839650-73839651	no coverage in NA17156	AT	Low Complexity	100	2
		chr11:73842034-73842037		TGTT	SINE	75	4
		chr11:73843420-73843423		TGTT		75	4
		chr11:73857248-73857256		ATACAAGGT		67	9
		chr2:223621493-223621497		AAAAA		100	5
		chr3:38588555-38588614		TATAAATACATATATGTATAATTATATAAATATATATTTTTATAAAGATATATTCATATG	LINE	92	60
		chr3:38589340-38589342		TAT	Simple Repeat	100	3
		chr21:34769887		A	LINE	100	1
		chr11:73839650-73839659	no coverage in NA17275	ATTAATTAAT	Low Complexity	100	10
		chr11:73842034-		TGTT	SINE	75	4

		73842037					
		chr11:73843420-73843423		TGTT		75	4
		chr2:223621489-223621498		TTAAAAAAAA		100	10
		chr3:38561031-38561035		AAAAA	Low Complexity	100	5
		chr3:38586364-38586366		TTC	LINE	67	3
		chr3:38588485-38588519		ATAGGTGCATATTATATAT TATAATATATACTTAT	LINE	86	35
		chr3:38588554-38588619		ATATAAATACATATATGTA TAATTATATAAATATATATT TTTATAAAGATATATTCAT ATGTGAAT	LINE	91	66
		chr3:38588657-38588668		AATATATTTTTT	LINE	100	12
		chr3:38589933-38589934		AA	LINE	100	2
		chr21:34661459		T	Low Complexity	100	1
		chr3:38589383		A	Low Complexity	100	1
		chr11:73839653-73839654	no coverage in NA17460	AA	Low Complexity	100	2
		chr11:73842034-73842037		TGTT	SINE	75	4
		chr11:73843419-73843423		TTGTT		80	5
		chr11:73844859-73844865		ATATATA	Simple Repeat	100	7
		chr11:73857248-73857256		ATACAAGGT		67	9
		chr2:223621491-223621492		AA		100	2
		chr3:38588493-38588511		ATATTATATATTATAATAT	LINE	100	19
		chr3:38588552-38588613		TTATATAAATACATATATG TATAATTATATAAATATAT ATTTTTATAAAGATATATT CATAT	LINE	94	62
		chr3:38588656-38588671		AAATATATTTTTTATA	LINE	100	16
		chr3:38589334-38589342		TTTATATAT	Simple Repeat	100	9
		chr3:38589382-38589384		TAT	Simple Repeat	100	3
		chr11:73839649-73839658	no coverage in NA17773	AATTAATTAA	Low Complexity	100	10
		chr11:73842034-73842037		TGTT	SINE	75	4
		chr11:73843420-73843424		TGTTA		80	5
		chr11:73857247-73857255		TATACAAGG		67	9
		chr3:38588493-38588496		ATAT	LINE	100	4
		chr3:38588552-38588576		TTATATAAATACATATATG TATAAT	LINE	92	25
		chr3:38588611-38588619		TATGTGAAT	LINE	78	9
		chr3:38588661-38588678		TATTTTTTATATTTATAT	LINE	100	18
		chr21:34779357		A	LINE	100	1

	low coverage	chr3:38588449-38588731	≤42 reads in ≥ 2 samples	ATTTATACATATAATAATATATAAATATGCACTTATATAGGTGCATATTATATATTATAATATATACTTATATTTATATATAGGTTTATATAATTGTATATAATTATATAAATACATATATGTATAATTATATAAATATATATTTTTATAAAGATATATTCATATGTGAATATATTTATATGGATTATATTTATATATTTAATATACAAATATATTTTTTATATTTATATATTTAATATACAAATACATTTTATATATTTATATATTTGTACATATATATTTA	LINE	91	283
		chr3:38589292-38589397		TATAATATATACACATGTAATATATAATATACACACATATATTTTATATATATATATATATTTGTGTGTGTGTGTGTATATATATACATATATATATATATATATT	SINE, Simple repeat	87	106
		chr21:34766415-34766484		TCCAAAAACTGATTTTAAAAATGAAGCCATGGGTGAAAGAAGTATTTAAAACTTCAAGAAAAATTACTTT	LINE	74	70
		chr21:34764611-34764675		TTAATAATTTTAATAGCCATTAAATTAACAAAATAATAATAATTAGATTTGGAATTTTTTAAAGA	Low complexity	88	65
		chr3:38609333-38609390		CTTCCCAATCCCAAGATCTTAAAGCTTTTTTGCTTGTTTTTATTCTAAAAACTTTAAA	LINE	72	58
		chr21:34763834-34763891		TATATATACATAAAATAGGAATACAAAACATGCTATTCCTATTATATGTGTAATAGGA	LINE	78	58
		chr3:38585386-38585442		TCTTAAAACAATTTTTGTGGTTTCATTGTTTTACATTACAATTTTAAAAATTAAATA	LINE	84	57
		chr21:34665968-34666016		ATCTTAAAAAGTTCTCATTATTTAAGAAAAAAAAATAAAAAGTTATAAT		88	49
		chr3:38588773-38588820		TATATTCTTTTATAGATTTTATATATTATATTTATGTGTATGAATATA	LINE	90	48
		chr21:34740239-34740285		CATTCCCTGCTCCGTCCGGACGGTGCCCACATTCCCTGCTCCGTCCG		32	47
		chr21:34769787-34769832		ATTTTCAGATTTCCAGAAAAAAAAAAGCAGAAAATCAATGTAACCT	LINE	74	46
		chr3:38584380-38584425		GGAGTTGTTCAAGATAAAAAAAAAGTAGGTGTATTGTATTTTTTTC	LINE	74	46
		chr2:223621480-223621521		ACATTTTTTTTAAAAAAAAAGTTTTATGAAGACTCAAAAGAA		83	42
		chr3:38588835-38588875		ACACACACATACACACACATTTTTTTTTTCTTTTTTTGAGAC	SINE	71	41
		chr7:150286114-150286152		CCCGGCGCGCATGGCCTCGATGTCGTCGGCCGACGAGGC		23	39
		chr21:34768590-34768628		AATTAACTAAAATTTCTTGGAACTAATAAGTGATTTTAT	LINE	82	39
		chr21:34769868-34769906		AAGCATGATTCATGAAAAAAATTTATAAATTATACTT	LINE	82	39

				GA			
		chr3:38563947-38563985		ATTCTGAAAATTTATGAAAAAAATTAAGAAAAAATTCTG	LINE	85	39
		chr3:38585314-38585351		TTCATTTAGTAAAATTTTTGATGTTACATTAAGAAAGC	LINE	79	38
		chr3:38586345-38586382		TAAATTAAGAAAATATATTTTCTTTCTTTTTTTTTTTT	LINE	92	38
		chr21:34792028-34792064		TGTTCATTTTTTAAATTCTTTTTTTTTCTTTTTTTTG	LINE	86	37
		chr11:73857232-73857265		GTATATTGTATACAATATACAAGGTGGGTATATT		74	34
		chr3:38586704-38586737		AGCACTATTTAGTTTTTAAAAAAATAATTTCTAG	SINE	82	34
		chr7:150288290-150288323		AATAATTTTTTCTTTTTAAAATTTTTTAACTTTT	SINE	94	34
		chr3:38610256-38610288		ATAATTTTTTCATATGAAATTTGCATATCTTTT	LINE	85	33
		chr21:34771485-34771517		CTTAATGAATGCAAATTTTGAAAAATTATTTAA	LINE	85	33
		chr3:38589915-38589946		GTTTTTTCTTTTTTTAAAAAAAATTTTCCCTC	LINE	81	32
		chr11:73839639-73839669		TATTTTAATTAATTAATTAATTTATTTTTTG	Low complexity	97	31
		chr11:73842457-73842487		TATATAGATATATAGATATATAGATATAGAT	Simple repeat	87	31
		chr3:38589536-38589565		ATGCCCAGCTAATTTTTAAAAATCTTTTTG	SINE	73	30
		chr11:73850460-73850488		TATGTACACATAGGTTTAAAAAAAAAAAA		83	29
		chr21:34760240-34760267		AAAAAAAAATGCTTAAGTATAAAATATT		89	28
		chr21:34766085-34766112		TATATATTTTTCTAGAAAAAAAACATGA		86	28
		chr21:34800133-34800160		TTTAACTACAGTATTTTTAAAATAGAAC		82	28
		chr21:34767518-34767545		AAGAAATAAGAAAAATTAATTAAAAATA	LINE	93	28
		chr21:34773303-34773330		TAAAATCTATTTTAAAAAATTTAAATAA	LINE	96	28
		chr11:73843778-73843803		TTTATTTTTATTTTTTCAAAATAGAA		92	26
		chr3:38561022-38561047		CTTTTTAAAAAAAAAAAAACAAAAAA	Low complexity	92	26
		chr21:34765892-34765916		AAATGAATTTTTGAAAAGAAAAATG	LINE	84	25
		chr21:34765980-34766004		CAAAATTTTAAAAACAAAAATTAGC	LINE	84	25
		chr21:34767323-34767347		AAATATTTTGAACTAAATGAAAATA	LINE	88	25
		chr11:73844845-73844868		TTAAATATATATATATATATATAT	Simple repeat	100	24
		chr11:73847952-73847975		ATTTTTTTTTTTTTTTTTTTTGAG	SINE	92	24
		chr11:73861890-73861913		TGTCTCAGGGGAAAAAAAAAAAAA	SINE	71	24
		chr21:34779348-34779371		AAAAATTAAAATTATTTTTAAAAA	LINE	100	24
		chr21:34661145-34661167		AAAAAAAAAAAAAAAAAAAAAAA	SINE	100	23
		chr21:34773600-34773622		AATAAGTATTAATTTATGAAAAT	LINE	91	23
		chr21:34794863-		CATATATAATCTTATTTTT	LINE	87	23

		34794885		CAAA			
		chr2:223618795-223618817		TTATTTTTTATTTTTTATTTTTT	SINE	100	23
		chr3:38656858-38656879		AGTTTAAAAAATGTGGCTGTTA		73	22
		chr11:73840545-73840566		TTTTTTTTTTTTTTTTTTTTTA	SINE	100	22
		chr21:34752232-34752253		TTAAATTTTATATTATTTATTT	Simple repeat	100	22
		chr21:34785307-34785328		AAAAAAAAAAAAAAAAAAAAAA	SINE	100	22
		chr11:73844432-73844452		TTTTTTTGAGACAGAGCCTTG	SINE	62	21
		chr21:34749348-34749368		CTTAAAAAAAAAAAAAAAGAA	LINE	90	21
		chr21:34754893-34754913		TTTTTTTCTTTTTTTTCTGAG	SINE	81	21
		chr21:34775648-34775668		CAAAAAAAAAAAAAAAAAAAA	SINE	95	21
		chr21:34781297-34781317		ATGTTGGTGTGCTGCACCCAT	LINE	48	21
		chr3:38651571-38651591		TATTTTAAAAATTAAAAAAAA	Low complexity	100	21
		chr21:34661327-34661346		TCAAAAAAAAAAAAAAAAAA		95	20
		chr21:34764325-34764344		TTCAAGTTGAATTTTTTATA		85	20
		chr11:73839006-73839025		TAGAAAAAAAAAAAAAAAAA	Simple repeat	95	20
		chr11:73839795-73839814		TTTTTTAAAAATTATTTTTA	SINE	100	20
		chr11:73840384-73840403		TTTTTTTTTTTTGAGACAGA	SINE	80	20
		chr21:34764556-34764575		ATAAAATAAAAATTTTGAAA	Low complexity	95	20
		chr21:34752368-34752386		AAAAAAATTTTGGCAGTCA		74	19
		chr21:34810583-34810601		GGTTTAAAAGCCTTTTAAA		74	19
		chr3:38610336-38610354		TGGAAACTTTAAAAATTTA	LINE	84	19
		chr21:34661453-34661471		ACTTTTTTATTTTAAAAAA	SINE, Low complexity	95	19
		chr21:34662769-34662787		AAAAAAACAAAAAATTTTT	SINE	95	19
		chr21:34774706-34774724		TTTTTTTTTTTTTTTTGAG	SINE	89	19
		chr21:34781207-34781225		TTTTTTTTTTAATATATAT	LINE	100	19
		chr21:34748682-34748699		TTTTAAAGAATTTTTTTA		94	18
		chr11:73842027-73842044		ATAGGGTTGTTATGAGGG	SINE	56	18
		chr21:34740384-34740400		CATTCCCTGCTCCGTCC		35	17
		chr21:34758606-34758622		AAGATTTAAAAATTGAG		82	17
		chr21:34668223-34668239		AAAAAAAAAAAAAAAAA	SINE	100	17
		chr21:34787644-34787660		TTTTTTTATTTTTTAAT	LINE	100	17
		chr3:38587375-38587391		TGAATTAAATAATTTAT	LINE	94	17
		chr3:38608142-		CAAAAAAAAAAAAATAG	SINE	88	17

		38608158					
		chr21:34738730-34738745		AAAAAAAAAAAAAAAA	SINE	100	16
		chr21:34789673-34789688		CTGGACTTTTTTTTTT	LINE	75	16
		chr21:34802359-34802374		TTTTTTTTTTTTTTTT	SINE	100	16
		chr3:38601417-38601432		TCTCAAAAAAAAAAAA	SINE	88	16
		chr11:73843414-73843428		TAGGGTTGTTATGAG		60	15
		chr21:34810697-34810711		TAAAAAAAATATTTT		100	15
		chr7:150283890-150283904		AGGCTCCCCCGCCCC		13	15
		chr11:73841785-73841799		TCAAAAAAAAAAAAT	SINE	93	15
		chr3:38575905-38575919		TTTTTTTTTTTTTTT	SINE	100	15
		chr3:38613625-38613639		CAACAAATATTTATT	SINE	87	15
		chr7:150286427-150286441		GCGCCCGCGCCGCCC	Low complexity	0	15
		chr21:34778470-34778483		GGAAAAAAAAAAAA		86	14
		chr11:73859080-73859093		AAAAAAAAAAATTA	SINE	100	14
		chr21:34768247-34768260		AAAAATCATATGAT	LINE	86	14
		chr21:34770609-34770622		AAAATCTGCATGTA	LINE	71	14
		chr21:34776430-34776443		AAAAAAAAAAAAAA	SINE	100	14
		chr21:34779857-34779870		AAAAAAAAAAAAAA	SINE	100	14
		chr21:34803622-34803635		TTAACTTTTTTTTT	SINE	93	14
		chr21:34813572-34813585		TTTTTTTTTTTTTT	SINE	100	14
		chr3:38636242-38636255		TTTTTCTTTTTTCT	SINE	86	14
		chr2:223625535-223625547		AAAAAAAAAAAAA		100	13
		chr11:73845021-73845033		TCAAAAAAAAAAA	SINE	92	13
		chr21:34771402-34771414		ATGTAAAATTTTA	LINE	92	13
		chr21:34790243-34790255		ACTTTTTTTTTTC	SINE, LINE	85	13
		chr7:150295303-150295315		AAATTAATTAATT	SINE	100	13
		chr21:34669909-34669920		CTGCGGGGCGCC		8	12
		chr21:34816357-34816368		AAAAAAAAAAAA		100	12
		chr11:73844590-73844601		TTTGTATTTTTT	SINE	92	12
		chr3:38619706-38619717		TTTTTTTTTTTT	SINE	100	12
		chr7:150277436-150277447		TTTTTTTTTTTT	Simple repeat	100	12
		chr3:38609269-38609279		TTTAACAATTT	LINE	91	11
		chr21:34788495-		AGGATTTTTTT	LINE	82	11

		34788505					
		chr2:223618906-223618916		CATTCTCCTGC	SINE	45	11
		chr3:38590562-38590572		TTTTTTTTTTT	SINE	100	11
		chr3:38610782-38610792		TTTTTTTTTGA	SINE	91	11
		chr3:38613419-38613428		GAAAAAAAAA		90	10
		chr21:34664204-34664213		TTTAAAAAAC	LINE	90	10
		chr21:34783162-34783171		AAAAAAAAAA	SINE	100	10
		chr11:73850436-73850444		TTTTTTTTA		100	9
		chr21:34741859-34741867		GGGGCAGCC		11	9
		chr11:73859245-73859253		AAAAAAAAA	SINE	100	9
		chr21:34738236-34738244		TAAAAATTA	SINE	100	9
		chr21:34771136-34771144		ACCAACATG	LINE	56	9
		chr21:34740875-34740882		AATTTTTT		100	8
		chr21:34750880-34750887		TTTGGAAA		75	8
		chr3:38579741-38579748		CAGGCCAA		38	8
		chr3:38616065-38616072		TTTTAAAT	LINE	100	8
		chr11:73858951-73858958		AAAAAAAA	SINE	100	8
		chr21:34658942-34658949		AAAAAAAA	SINE	100	8
		chr21:34764767-34764774		GAAATTTT	LINE	88	8
		chr3:38589750-38589757		TTTTTTTG	LINE	88	8
		chr3:38644238-38644245		AAAAAAAA	SINE	100	8
		chr21:34789626-34789632		CAATTCT	LINE	71	7
		chr3:38556661-38556667		TTTTTTT	SINE	100	7
		chr3:38563736-38563742		AAATAAA	LINE	100	7
		chr3:38588256-38588262		TTATATA	Low complexity	100	7
		chr7:150289324-150289330		TTTTTTT	SINE	100	7
		chr21:34799621-34799626		TTTTTG		83	6
		chr21:34774288-34774293		AAAAAA	SINE	100	6
		chr21:34665956-34665960		TGCAG		40	5
		chr2:223618295-223618299		TTTAA		100	5
		chr7:150282296-150282300		AAAAA		100	5
		chr21:34764804-34764808		TAAAA	LINE	100	5
		chr21:34817959-		TTTTT	SINE	100	5

		34817963					
		chr7:150291368-150291372		CCAGA	SINE	40	5
		chr21:34743183-34743186		TGCT		50	4
		chr21:34660979-34660982		TGTA	SINE	75	4
		chr21:34739279-34739282		TCTA	SINE	75	4
		chr21:34764859-34764862		AACA	LINE	75	4
		chr2:223622088-223622091		TAAA	LINE	100	4
		chr21:34815282-34815284		TTA		100	3
		chr21:34767716-34767718		TTA	LINE	100	3
		chr21:34794247-34794249		TAA	Low complexity	100	3
		chr7:150275826-150275828		CCG	Low complexity	0	3
		chr21:34748441-34748442		AA		100	2
		chr21:34815564-34815565		AT		100	2
		chr7:150294314-150294315		AA		100	2
		chr21:34764814-34764815		TA	LINE	100	2
		chr21:34814581		A		100	1
		chr2:223625484		T		100	1
		chr3:38606725		A		100	1
		chr11:73847538		A	SINE	100	1
		chr21:34662447		T	Simple repeat	100	1
		chr21:34746912		T	SINE	100	1
		chr21:34766497		A	LINE	100	1
		chr21:34802205		T	SINE	100	1
		chr2:223622081		T	LINE	100	1
		chr3:38587759		T	SINE	100	1

Supplemental Table 7. Analysis of 23 LR-PCR amplicons for imbalanced allelic amplification. The distribution of the alternate allele read frequency at positions known to be heterozygous based on ABI Sanger data is shown with the data from all three technologies combined. Amplicons that do not follow the expected AARF distribution from Supplemental Figure 1 (with $p < 0.01$) are highlighted in grey and were removed from subsequent analysis.

Amplicon name	Amplicon coordinate	Sample	p-value	# of Sanger Het positions by 3 NGS	Distribution of AARF						
					0-10	10-20	20-40	40-60	60-80	80-90	90-100
LPCR_382423_11004	chr2:223616214-223627218	NA17156		3*2	0	0	1	3	2	0	0
		NA17460		3*6	0	1	5	10	2	0	0
		NA17275		3*8	3	1	7	12	1	0	0
		NA17773		3*3	0	0	4	5	0	0	0
LPCR_721496_11101	chr3:38553978-38565079	NA17773		3*1	0	0	1	2	0	0	0
LPCR_721498_8720	chr3:38575485-38584205	NA17156		3*1	0	0	2	1	0	0	0
LPCR_721500-_11083	chr3:38595323-38606406	NA17275	10^{-7}	3*2	6	0	0	0	0	0	0
		NA17773		3*1	0	0	0	3	0	0	0
LPCR_721501_11969	chr3:38606353-38618322	NA17275		3*1	0	0	0	3	0	0	0
LPCR_721502_11967	chr3:38618395-38630362	NA17156		3*11	0	0	9	22	2	0	0
		NA17460		3*3	0	0	3	6	0	0	0
		NA17275		3*12	1	1	2	31	1	0	0
		NA17773		3*14	2	1	10	27	2	0	0
LPCR_721503_11821	chr3:38630330-38642151	NA17156		3*1	1	0	1	1	0	0	0
		NA17460		3*4	0	0	1	9	2	0	0
		NA17275		3*4	1	0	3	7	1	0	0
		NA17773		3*1	0	0	0	3	0	0	0
LPCR_721504_11891	chr3:38642100-38653991	NA17156		3*3	0	0	2	7	0	0	0
		NA17460		3*7	0	0	7	13	1	0	0
		NA17275		3*3	0	0	6	3	0	0	0
		NA17773		3*2	0	0	2	1	3	0	0
LPCR_721505_11811	chr3:38653478-38665289	NA17156		3*3	0	0	4	2	3	0	0
		NA17460		3*4	0	0	5	7	0	0	0
		NA17275		3*5	0	0	5	10	0	0	0
		NA17773		3*7	0	0	6	13	2	0	0
LPCR_54275_11143	chr7:150269610-150280753	NA17156	0.0001	3*1	3	0	0	0	0	0	0
		NA17460		3*6	5	1	3	8	1	0	0
		NA17773		3*8	3	0	7	12	1	1	0
LPCR_54276_4905	chr7:150280713-150285618	NA17156		3*3	0	0	3	4	2	0	0
		NA17275		3*2	0	0	1	5	0	0	0
		NA17773		3*2	0	0	0	4	2	0	0
LPCR_1527027_3087	chr7:150284619-150287706	NA17156		3*3	0	0	2	7	0	0	0
		NA17460		3*3	0	0	3	5	1	0	0
		NA17275		3*1	0	0	0	2	0	1	0
		NA17773		3*3	0	0	3	5	1	0	0
LPCR_54277_11487	chr7:150286689-150298176	NA17156		3*1	0	0	1	2	0	0	0
		NA17460		3*3	0	0	3	5	1	0	0

		NA17275		3*2	0	0	1	5	0	0	0
		NA17773		3*2	0	0	1	5	0	0	0
LPCR_54278_3423	chr7:150298170-150301593	NA17156		3*2	0	0	1	5	0	0	0
		NA17460		3*2	0	0	4	1	1	0	0
		NA17275		3*2	1	0	2	3	0	0	0
		NA17773		3*4	3	0	3	6	0	0	0
LPCR_240580_9001	chr11:73836950-73845951	NA17156	$< 10^{-16}$	3*13	0	0	1	2	22	10	4
		NA17460		3*9	2	1	5	10	9	0	0
		NA17275		3*9	1	0	2	11	13	0	0
		NA17773	$2*10^{-9}$	3*10	0	0	1	4	21	4	0
LPCR_240581_8458	chr11:73845929-73854387	NA17156		3*2	0	0	0	6	0	0	0
		NA17460		3*2	0	0	1	5	0	0	0
		NA17275		3*3	0	0	2	5	2	0	0
		NA17773		3*3	0	0	4	4	1	0	0
LPCR_1977_14227	chr21:34657259-34671486	NA17460	0.0008	3*9	18	0	0	0	0	0	9
		NA17275	$3*10^{-7}$	3*8	6	0	0	0	1	6	11
		NA17773	0.002	3*9	16	1	1	0	0	0	9
LPCR_1983_10796	chr21:34735911-34746707	NA17156		3*7	0	0	4	15	2	0	0
		NA17460		3*2	0	0	2	3	1	0	0
		NA17773		3*4	1	1	3	7	0	0	0
LPCR_778623_3341	chr21:34746238-34749579	NA17460		3*1	0	0	1	1	1	0	0
		NA17773		3*2	0	0	1	5	0	0	0
LPCR_778624_11646	chr21:34749486-34761132	NA17156		3*4	0	0	2	8	2	0	0
		NA17460		3*1	0	0	1	2	0	0	0
		NA17773		3*2	0	0	1	5	0	0	0
LPCR_1988_4624	chr21:34795398-34800022	NA17156		3*1	0	0	1	1	1	0	0
		NA17460		3*1	0	0	1	2	0	0	0
LPCR_1989_6410	chr21:34799969-34806379	NA17156		3*2	0	0	2	3	1	0	0
		NA17460		3*1	0	0	1	2	0	0	0
LPCR_1990_12103	chr21:34806347-34818450	NA17156		3*5	0	0	0	14	1	0	0
		NA17460		3*2	1	0	1	4	0	0	0

Supplemental Table 8. Accuracy of NGS and Sanger technologies assessed by genotyping SNPs in the Illumina Human Hap550 BeadChip.

	NA17156	NA17275	NA17460	NA17773	All Individuals Combined
ABI Sanger	100% (24/24)	96% (24/25)	96% (23/24)	100% (22/22)	98% (93/95)
Roche 454	99% (76/77)	97% (75/77)	98% (78/80)	96% (76/79)	97.4% (305/313)
Illumina GA	100% (77/77)	100% (77/77)	100% (80/80)	100% (79/79)	100% (313/313)
ABI SOLiD	100% (77/77)	100% (77/77)	100% (80/80)	99% (78/79)	99.7% (312/313)

Supplemental Table 9. Discordant genotypes (in bold) between SNPs from the Illumina HumanHap550 array and the sequencing technologies.

Individual	Location	dbSNP rs id	**HumanHap550 Genotype**	ABI Sanger Genotype	Roche 454 Genotype	Illumina GA Genotype	ABI SOLiD Genotype
NA17275	chr11:73843968	rs7789	**GT**	**TT**	GT	GT	GT
NA17460	chr11:73843968	rs7789	**GT**	**TT**	GT	GT	GT
NA17773	chr11:73861481	rs633335	**CC**	-	**CT**	CC	CC
NA17275	chr21:34743289	rs2070357	**CC**	CC	**CT**	CC	CC
NA17773	chr21:34743289	rs2070357	**CT**	CT	**TT**	CT	CT
NA17773	chr21:34761833	rs11701051	**CT**	-	CT	CT	**TT**
NA17460	chr21:34806395	rs11702354	**AG**	AG	**GG**	AG	AG
NA17773	chr21:34806395	rs11702354	**AA**	AA	**GG**	AA	AA
NA17275	chr3:38606427	rs7373157	**GT**	-	**GG**	GT	GT
NA17156	chr3:38630647	rs9856587	**CT**	CT	**CC**	CT	CT
NA17460	chr3:38635940	rs12498069	**AC**	-	**CC**	AC	AC

Supplemental Table 10. Comparison of ABI Sanger platform base calls at 258,879 bp (4 samples) sequenced by 3 NGS Technologies.

		Sanger			
		Ref	Het	Alt	N/N
Roche 454	Ref	258,290	12	1	98
	Het	16	203	6	14
	Alt	1	1	104	2
	N/N	131	0	0	0
Illumina GA	Ref	258,388	3	0	96
	Het	28	217	0	15
	Alt	3	0	111	3
	N/N	15	0	0	0
ABI SOLiD	Ref	257,919	6	0	92
	Het	28	200	3	13
	Alt	8	6	108	1
	N/N	480	8	0	7

Supplemental Table 11. Quality metrics for the four DNA Samples in the three NGS technologies when compared to ABI Sanger data (Figure 4).

		Sequencing Accuracy	Variant Accuracy	Coverage Rate	False Positive Rate	False Negative Rate	Variant Discrepancy Rate
Roche 454	ALL	0.99990	0.948	0.9996	0.025	0.031	0.022
	NA17156	0.99990	0.956	0.9994	0.022	0.011	0.034
	NA17275	0.99988	0.919	0.9999	0.033	0.048	0.034
	NA17460	0.99990	0.946	1.0000	0.022	0.032	0.022
	NA17773	0.99993	0.962	0.9990	0.026	0.038	0.000
Illumina GA	ALL	0.99991	1.000	1.0000	0.063	0.000	0.000
	NA17156	0.99992	1.000	1.0000	0.053	0.000	0.000
	NA17275	0.99990	1.000	1.0000	0.086	0.000	0.000
	NA17460	0.99994	1.000	1.0000	0.041	0.000	0.000
	NA17773	0.99990	1.000	0.9999	0.080	0.000	0.000
ABI SOLiD	ALL	0.99985	0.963	0.9984	0.078	0.009	0.028
	NA17156	0.99983	0.967	0.9979	0.072	0.000	0.033
	NA17275	0.99990	0.984	0.9987	0.076	0.000	0.016
	NA17460	0.99982	0.921	0.9984	0.065	0.034	0.047
	NA17773	0.99985	0.988	0.9985	0.101	0.000	0.013

Supplemental Table 12. Discordant genotypes between NGS platforms and ABI Sanger

Found by		NCBI36_sample	Upstream Sequence	Downstream Sequence	Repeats	ABI Sanger genotype	NGS genotypes			Category
							Roche 454	Illumina GA	ABI SOLiD	
False Positive	Roche 454 only	chr21_34774913_NA17275	GTTTTGCCTTGTTAGCCAAGCTGGTCTC	AACTCCTGACCTCAGGTGATCCACCTGCTT	AluSx	GG	AG	GG	GG	repetitive element & in/del
		chr21_34774913_NA17773	GTTTTGCCTTGTTAGCCAAGCTGGTCTC	AACTCCTGACCTCAGGTGATCCACCTGCTT	AluSx	GG	AG	GG	GG	repetitive element & in/del
		chr2_223627087_NA17275	GGTGGTTCAGGAGACTCTTCCTGATCTT	CTAGAAGGGGTAAAGTGGGGTTGAACAAGG		TT	CT	TT	TT	
		chr3_38602150_NA17156	ATTGGATGGGTGGGTAGCTGGGTAGATG	GTGGATGGTGTGTGTGTGGCCCTTGGCCAA	(TGGA)n	AA	AT	AA	AA	simple repeat & in/del
		chr3_38602150_NA17460	ATTGGATGGGTGGGTAGCTGGGTAGATG	GTGGATGGTGTGTGTGTGGCCCTTGGCCAA	(TGGA)n	AA	AT	AA	AA	simple repeat & in/del
		chr3_38602150_NA17773	ATTGGATGGGTGGGTAGCTGGGTAGATG	GTGGATGGTGTGTGTGTGGCCCTTGGCCAA	(TGGA)n	AA	AT	AA	AA	simple repeat & in/del
	Illumina GA only	chr11_73842453_NA17275	GATACAGAGATACAGAGATACAGAGATA	AGATATATAGATATATAGATATATAGATAT	(CAGAGA)n	CC	CC	CT	CC	simple repeat
		chr11_73845036_NA17275	TAGGGAGACTGTCTCAAAAAAAAAAAAA	AAAAGAAAAGAAAAGAAGTTTCTCACATCA	AluSx	GG	GG	AG	GG	homopolymer
		chr21_34749366_NA17156	TTCCCTGGAACTTAAAAAAAAAAAAAAA	AAACAAAAACAACCAACCAAAAATATATCT	L1PA10	GG	GG	AG	GG	homopolymer & in/del
		chr21_34749366_NA17275	TTCCCTGGAACTTAAAAAAAAAAAAAAA	AAACAAAAACAACCAACCAAAAATATATCT	L1PA10	GG	GG	AG	GG	homopolymer & in/del
		chr21_34749366_NA17460	TTCCCTGGAACTTAAAAAAAAAAAAAAA	AAACAAAAACAACCAACCAAAAATATATCT	L1PA10	GG	GG	AG	GG	homopolymer & in/del
		chr21_34749366_NA17773	TTCCCTGGAACTTAAAAAAAAAAAAAAA	AAACAAAAACAACCAACCAAAAATATATCT	L1PA10	GG	GG	AG	GG	homopolymer & in/del
		chr3_38563723_NA17275	TGTGCTGTGGGAGACAGTATGGCAGTTC	TAAAAAAATTAAAAATAAAATTGCCATATG	L1MB3	CC	CC	AC	CC	repetitive element
		chr3_38564670_NA17156	ACAACATCTGGCACTTGATGTCTGTCCT	CCCTCCTGCTGGTCATTGTGCAGTTCTGGA		TT	TT	CT	NN	in/del
		chr7_150279372_NA17773	ATGGTGGCCCCTGGAGTCTCTAAGTTCC	GGGCCTCACTCTGGCCCGGCTAGCAGCCTC		AA	AA	AG	AA	in/del
		chr7_150286149_NA17773	CGCATGGCCTCGATGTCGTCGGCCGACG	GGCGCGGCGCACGCTGGCGCAGCTTTCTCG		AA	AA	AG	AA	low coverage
		chr7_150298286_NA17460	GGGGAGAAAAGAGGCCCAGTCTCCCCCC	CACACACCCCCAAGCTGGCCCTTCTGCTGC		TT	TT	CT	TT	homopolymer
	ABI SOLiD only	chr11_73842038_NA17460	GAATACCTACTTACTACATAGGGTTGTT	TGAGGGTTTTAAAAGCTAAATGAATTCAAG	MIR	AA	AA	AA	CC	repetitive element
		chr11_73843792_NA17460	AGAACCCAAAGGCTTTTATTTTTATTTT	TCAAAATAGAAGTACCTTTTTTCCTTTTAT		TT	TT	TT	CT	
		chr21_34741405_NA17275	CTAAGAAGGGTCTGGCTTACACAGGGGC	ATGAGACGTGGCAGGCATAGCTGGGCTGCT		CC	CC	CC	TT	
		chr21_34741865_NA17773	ACGGAGGCCCTCTCTATCTGCTGGGGCA	CCCTCCGGGTGCCCCGCTGGAAGGCAGAGC		GG	GG	GG	CG	in/del
		chr21_34749352_NA17275	ACATCCTGCACATGTTCCCTGGAACTTA	AAAAAAAAAAAAAGAAACAAAAACAACCAA	L1PA10	AA	AA	AA	AT	homopolymer & in/del
		chr21_34774705_NA17156	AGAAAATGAAATTGAGGAGGGGGACTCA	TTTTTTTTTTTTTTTTGAGACAGAGTCTCA	AluSx	CC	CC	CC	CT	homopolymer & in/del
		chr21_34807310_NA17460	CCAAGATCAATGTTTTTTTCCTTTATCC	GATTTTAAAATCAAGTACACTTTATTGAGG		TT	TT	TT	CT	
		chr21_34807310_NA17773	CCAAGATCAATGTTTTTTTCCTTTATCC	GATTTTAAAATCAAGTACACTTTATTGAGG		TT	TT	TT	CT	
		chr3_38589927_NA17773	TTGCTGTGAGATCTCTGTTTTTTCTTTT	TTAAAAAAAATTTTCCCTCAGCAATTTGGA	L1MEe	TT	TT	TT	CT	homopolymer
		chr3_38596373_NA17156	GCACTCCCGGCTTGCGTGCGCGCACGCG	GCGCACACACACACACACACCCCCACACAC	(CA)n	CC	CC	CC	TT	simple repeat & in/del
		chr3_38602169_NA17773	GGGTAGATGAGTGGATGGTGTGTGTGTG	CCCTTGGCCAACTTACCACAAGGTTGCCAA		GG	GG	GG	GT	simple repeat & in/del
		chr3_38643193_NA17156	TCAGATCCCAGGGAGTTTAAAAAAAAAA	CCACCCAAAGAAAAGGTAATTAACTCTGGA		AA	AA	AA	AC	homopolymer
		chr3_38645101_NA17156	TAGAGAGGCCACACCCCCGGAGTGAGGC	AGAGACGGGCTCAGAGCAAGAAAAGCTGGC		CC	CC	CC	TT	
		chr7_150276467_NA17460	CCCCTTCCTCCCCTCCCCCGCCTCACCC	TGTCCGTGCGCCTGCGGAAGGACAACTTGC		TT	GT	GT	GG	

		chr7_150279376_NA17773	TGGCCCCTGGAGTCTCTAAGTTCCAGGG	CTCACTCTGGCCCGGCTAGCAGCCTCAGTT		CC	CC	CC	CG	in/del
		chr7_150290648_NA17156	ACTCACACTTACACACACACACGAGAGA	AGAGAGAGAGAGAGAGAGAGAGAGAGGAGT	(GA)n	CC	CC	CC	CG	simple repeat & in/del
		chr7_150290648_NA17275	ACTCACACTTACACACACACACGAGAGA	AGAGAGAGAGAGAGAGAGAGAGAGAGGAGT	(GA)n	CC	CC	CC	CG	simple repeat & in/del
		chr7_150290648_NA17460	ACTCACACTTACACACACACACGAGAGA	AGAGAGAGAGAGAGAGAGAGAGAGAGGAGT	(GA)n	CC	CC	CC	CG	simple repeat & in/del
	Roche 454 and Illumina GA	chr21_34741860_NA17156	GCAGAACGGAGGCCCTCTCTATCTGCTG	GGCAGCCCTCCGGGTGCCCCGCTGGAAGGC		GG	CG	CG	NN	in/del
		chr7_150276467_NA17460	CCCCTTCCTCCCCTCCCCCGCCTCACCC	TGTCCGTGCGCCTGCGGAAGGACAACTTGC		TT	GT	GT	GG	
	Illumina GA and ABI SOLiD	chr2_223624619_NA17773	ATAATCCTACCTGCAGAAGACCCCCCCC	CCGCCAGGCACAACGATTTTACAGACGAGG		CC	CC	CG	CG	homopolymer & in/del
		chr3_38564670_NA17275	ACAACATCTGGCACTTGATGTCTGTCCT	CCCTCCTGCTGGTCATTGTGCAGTTCTGGA		TT	TT	CT	CT	simple repeat & in/del
		chr3_38564670_NA17460	ACAACATCTGGCACTTGATGTCTGTCCT	CCCTCCTGCTGGTCATTGTGCAGTTCTGGA		TT	TT	CT	CT	simple repeat & in/del
		chr3_38564670_NA17773	ACAACATCTGGCACTTGATGTCTGTCCT	CCCTCCTGCTGGTCATTGTGCAGTTCTGGA		TT	TT	CT	CT	simple repeat & in/del
		chr3_38596386_NA17773	GCGTGCGCGCACGCGCGCGCACACACAC	CACACACCCCCACACACACCACACACACCC	(CA)n	AA	AA	AC	AC	simple repeat & in/del
		chr3_38596396_NA17156	ACGCGCGCGCACACACACACACACACCC	CACACACACCACACACACCCAGGAGCCAGA	(CA)n	CC	CC	AA	AA	simple repeat & in/del
		chr3_38596400_NA17156	GCGCGCACACACACACACACACCCCCAC	CACACCACACACACCCAGGAGCCAGAGTCA	(CA)n	AA	AA	CC	CC	simple repeat & in/del
		chr3_38638394_NA17275	TTACTGAAGTGGGGATAGCCCTGGGGTA	CTCCTAGCTGCCTACCTCCTAGCTGCCTAC	MIR	CC	CC	CG	CG	repetitive element & in/del
		chr3_38638394_NA17773	TTACTGAAGTGGGGATAGCCCTGGGGTA	CTCCTAGCTGCCTACCTCCTAGCTGCCTAC	MIR	CC	CC	CG	CG	repetitive element & in/del
	All 3 NGS	chr11_73843968_NA17275	TCAGAGTTCTGCTATACACAAATACGCA	GTTTCAATGGGTACTTCCAGGGACCGCATG		TT	GT	GT	GT	
		chr11_73843968_NA17460	TCAGAGTTCTGCTATACACAAATACGCA	GTTTCAATGGGTACTTCCAGGGACCGCATG		TT	GT	GT	GT	
		chr21_34759676_NA17460	ATGGGTAGGAAAGAACACTAAATCTACT	GCACAATGTTTGATATTTAAAGATAAACAT		CC	CT	CT	CT	
		chr21_34807876_NA17156	ATCTATCCAATCCACTTGCGTGTTGAAC	TCTACTATGAGCCATTGGCTACCAGGAATC		GG	AG	AG	AG	
		chr3_38596474_NA17156	GGAACAGAGAAGGGAAATGAGAGACAGC	GGAATTCCCCTCCTGTGCTGGAGATCATTG		CC	TT	TT	TT	
		chr3_38639168_NA17156	CAATCCCCCTCCACCCAGGCTGGTCTCT	TCCCTTCCCCGGCACTTGCAGCTGGCCCCC		TT	GT	GT	GT	
		chr7_150284380_NA17773	CAGGCGGTGGGAGCAGGGCCAGGAGCCC	CCGAGGTAGCAGGGCAGCTCCCTTACATGA		GG	AG	AG	AG	
		chr7_150293045_NA17773	CAAGAGGCCAACCAGTGAATGAGAGAGA	GGAGGGAGAGAGACAGAGACAGGGAGAGAG	GA-rich	AA	AG	AG	AG	
		chr7_150293190_NA17156	CAGGTTGAGGGGCCCCAAGCTCAGACCC	GACCCCACAGAAGCAGCATCTCCAGACAGA		TT	GT	GT	GT	
False Negative	Roche 454 only	chr11_73843069_NA17275	TAACTCACAACCAAAATAATTTGTATTA	TTTATGATTATGCCCATGGAGTGTGAGGAC		AT	TT	AT	AT	in/del
		chr11_73845036_NA17460	TAGGGAGACTGTCTCAAAAAAAAAAAAA	AAAAGAAAAGAAAAGAAGTTTCTCACATCA	AluSx	AG	GG	AG	NN	homopolymer
		chr21_34743289_NA17773	CAGTGAAACAGTGTCATTAATCCACCCC	CACCCCTTACAACAGCCAAAAAGAAATCCA		CT	TT	CT	CT	
		chr21_34806395_NA17460	AGTGTCAAATGCGTTGTTCCCTGGAGAG	AAGGCTTATTTCCAGAAAGTACTCTGACGT		AG	GG	AG	AG	
		chr21_34806395_NA17773	AGTGTCAAATGCGTTGTTCCCTGGAGAG	AAGGCTTATTTCCAGAAAGTACTCTGACGT		AA	GG	AA	AA	In/del
		chr3_38622646_NA17773	TAGATCTTCCCTGCGGACCTGAGGGTCT	GGGGAGCAAGGGGGCAGAGGTCACCCTCAC		GT	GG	GT	GT	
		chr3_38630647_NA17156	CCCGACCAGCCCTCTCCCTTGGCCCCCT	CCACGGGGGTGGGGGCAGTGCTGTGGATAG		CT	CC	CT	CT	in/del
		chr3_38633238_NA17275	CAGGGGCAGAAAGAGCAAAGGGTGGTGG	TGAGAATCCTATCCCATCACCAGCCCCACT		GT	GG	GT	GT	in/del
		chr7_150275327_NA17460	CTGGACCAGACTCCAGGGCGTGCCCCCC	ACCCCACCTGCACTCCCTCACCTGTTGAGC		CT	CC	CT	CT	homopolymer
		chr7_150293045_NA17275	CAAGAGGCCAACCAGTGAATGAGAGAGA	GGAGGGAGAGAGACAGAGACAGGGAGAGAG	GA-rich	AG	AA	AG	AG	simple repeat
	ABI SOLiD only	chr11_73844514_NA17460	TCTTGGCTTACTGCAACCTCCACCTCCC	GGTTCAAACAATTCTCCTGCCTCAGCCTCC	AluSx	AG	AG	AG	GG	repetitive element
		chr7_150278902_NA17460	AGAGGTTCCCCTCTGCCACCCCACTCTT	CCAGCCTGCCACCCACTGGCCACGCTCTGG		CT	CC	CT	CC	adjacent variants

		chr7_150278903_NA17460	GAGGTTCCCCTCTGCCACCCCACTCTTC	CAGCCTGCCACCCACTGGCCACGCTCTGGT		CG	CC	CG	CC	adjacent variants
	All 3 NGS	chr7_150299914_NA17773	CTGTGAGGACTGGCAGAGGAGGAGCCAC	GCCCCCATGGCGATGTGGGAGCACAGGTGT		AC	AA	AA	AA	
		chr7_150275444_NA17773	TGGAGAGGGGGATGTTGAGGAGGCTGGG	GTGGGGGCGGGGCATCGAGGGAGCTCCTGG		AG	GG	GG	GG	
		chr2_223624557_NA17275	CAGGCAGCCTTCAATCTTAGAAGGGTTA	GCTGAAAGGGTCTCAAAAGGTCACGTGGTT		AG	AA	AA	AA	
Variant Discrepant Calls	Roche 454 only	chr21_34743289_NA17275	CAGTGAAACAGTGTCATTAATCCACCCC	CACCCCTTACAACAGCCAAAAAGAAATCCA		CC	CT	CC	CC	
		chr3_38643196_NA17156	GATCCCAGGGAGTTTAAAAAAAAAAACC	CCCAAAGAAAAGGTAATTAACTCTGGAGGG		GG	AG	GG	GG	
		chr7_150279919_NA17460	GGGCAAGGGGGGCAAGGGAGGAGGGGAG	TGCTGCGGCCCTCAGAGCGAGCATCAGAGG		CC	CG	CC	CC	
		chr7_150286470_NA17275	GCGCCGCCCGACCGCACCGACGACTCCC	GGCCGTCAGCGCCAGCAGCGCGGGCAGCTT		AG	AA	AG	NN	
		chr7_150293045_NA17156	CAAGAGGCCAACCAGTGAATGAGAGAGA	GGAGGGAGAGAGACAGAGACAGGGAGAGAG	GA-rich	GG	AG	GG	GG	simple repeat
		chr7_150293045_NA17460	CAAGAGGCCAACCAGTGAATGAGAGAGA	GGAGGGAGAGAGACAGAGACAGGGAGAGAG	GA-rich	GG	AG	GG	GG	simple repeat
	ABI SOLiD only	chr11_73842457_NA17460	CAGAGATACAGAGATACAGAGATACAGA	ATATAGATATATAGATATATAGATATAGAT	(TA)n	GG	GG	GG	GT	simple repeat
		chr11_73843069_NA17460	TAACTCACAACCAAAATAATTTGTATTA	TTTATGATTATGCCCATGGAGTGTGAGGAC		AT	AT	AT	AA	
		chr11_73845278_NA17275	CAACATATTAATGTGGCAGTATATTTTC	TAATGTTTGCAAAAGTAAGATATTTTAGAA	Arthur1	AG	AG	AG	GG	repetitive element
		chr11_73845278_NA17460	CAACATATTAATGTGGCAGTATATTTTC	TAATGTTTGCAAAAGTAAGATATTTTAGAA	Arthur1	AG	AG	AG	GG	repetitive element
		chr21_34746100_NA17460	GTCCTGTCAGAGACTGGCCCCTGCCTCA	TGAGCTGAGTGGCAACACCACTCACTCCAA		CT	CT	CT	CC	
		chr3_38592130_NA17156	AGGGCCGTGGGCACCACCCGTGGGGGCC	CACGCTCTGCGGGCACTCTTTGAGGAGGAT		GG	GG	GG	CG	
		chr7_150279131_NA17773	TGGCCGACACGTTGCCGAAGATGCTAGC	TACATGAGGGCTGGGGGCGTGGGCACGTGG		AG	AG	AG	GG	
		chr7_150283286_NA17156	AGGAGCCAGGCGCGGGTGAGCAGGGCCA	GCCCCACGGCTCCCAAAGCTTCCTACTTCC		AG	AG	AG	AA	
	Roche 454 and ABI SOLiD	chr7_150298282_NA17156	CTCTGGGGAGAAAAGAGGCCCAGTCTCC	CCCTCACACACCCCCAAGCTGGCCCTTCTG		TT	CT	TT	CT	in/del

Supplemental Table 13. Analysis of eleven heterozygous indels identified by ABI Sanger. Allele1 and **Allele2** refer to the base calls detected at the locus. **Homopolymer Length** is the length of the homopolymeric region flanking the reported indel.

**ABI SANGER**						**Roche 454**		
**NCBI36**	**Sample**	**Allele1**	**Allele2**	**Homopolymer Length**	**resolved by 454**	**NCBI36**	**Allele1**	**Allele2**
chr21_34795794	NA17773	-	T	5	no			
chr21_34806868	NA17156	-	T	6	no			
chr2_223624624	NA17773	-	GCC		yes	chr2_223624624	-	GCC
chr3_38633475	NA17156	-	GAGGGCTGCAAGCTG		yes	chr3_38633476	-	GAGGGCTGCAAGCTG
						chr3_38633465	A	-
chr3_38633475	NA17275	-	GAGGGCTGCAAGCTG		partial	chr3_38633469	CTG	GCAA
chr3_38633475	NA17460	-	GAGGGCTGCAAGCTG		yes	chr3_38633476	-	GAGGGCTGCAAGCTG
chr3_38638411	NA17275	CCTCCTAGCTGCCTAC	G		yes	chr3_38638394	CCTCCTAGCTGCCTAC	G
chr3_38638411	NA17773	CCTCCTAGCTGCCTAC	G		yes	chr3_38638394	CCTCCTAGCTGCCTAC	G
chr7_150282302	NA17460	-	A	11	no			
chr7_150282302	NA17773	-	A	11	no			
chr7_150299420	NA17156	-	G	6	no			

Supplemental Table 14. Forty three additional indels not found by ABI Sanger and identified by Roche 454 in the 88-kb overlapping ABI Sanger sequences

Coord NCBI36	Sample	alt/ref	Total Reads	Alt reads	upstream sequence	downstrem sequence	repeat
chr11_73841786	NA17460	-/A	23	8	GATTAGGCCACTGCACTCCAGCCTGAGCCACAGAGCGAGACCCTGTCT	AAAAAAAAAAAAATTCTGCCTCAAGGTCTGTTTCTAGGAAACCCAACTTCA	AluJo
chr21_34742194	NA17773	T/-	24	15	GGTTTGTGGCTGTGTCACTGCACCCTCTGCCTCTGTCACGGCATGGTG	CCTCCCTATGCATCTGTGTCTGAATTTCCCTCTTCCGATAAGGACTCCAG	MLT1C
chr21_34749371	NA17275	A/-	11	5	CACATCCTGCACATGTTCCCTGGAACTTAAAAAAAAAAAAAAAGAAAC	AAAACAACCAACCAAAAATATATCTAAAATGTCATCTGTTAGCAATTGAC	
chr21_34774705	NA17156	C/-	25	14	TATCTCTGCAAAGAGAGGAAAGAAAATGAAATTGAGGAGGGGGACTCA	TTTTTTTTTTTTTTTTGAGACAGAGTCTCACTCTATTGCCCAGGCTGGAG	AluSx
chr21_34774890	NA17275	GCCT/CACCA	17	4	TACAGGCGCACACCACCACACCTGTATTTTTTGTAGAGATGGAGTTTT	CCTTGTTAGCCAAGCTGGTCTCGAACTCCTGACCTCAGGTGATCCACCTG	AluSx
chr21_34774898	NA17275	AGCCAA/GGTCAG	17	4	CACACCACCACACCTGTATTTTTTGTAGAGATGGAGTTTTGCCTTGTT	GCCAAGCTGGTCTCGAACTCCTGACCTCAGGTGATCCACCTGCTTTGGTC	AluSx
chr21_34774884	NA17773	A/-	12	3	TGAGACTACAGGCGCACACCACCACACCTGTATTTTTTGTAGAGATGG	GTTTTGCCTTGTTAGCCAAGCTGGTCTCGAACTCCTGACCTCAGGTGATC	AluSx
chr21_34774890	NA17773	GCCT/CACCA	13	4	TACAGGCGCACACCACCACACCTGTATTTTTTGTAGAGATGGAGTTTT	CCTTGTTAGCCAAGCTGGTCTCGAACTCCTGACCTCAGGTGATCCACCTG	AluSx
chr21_34774898	NA17773	AGCCAA/GGTCAG	15	5	CACACCACCACACCTGTATTTTTTGTAGAGATGGAGTTTTGCCTTGTT	GCCAAGCTGGTCTCGAACTCCTGACCTCAGGTGATCCACCTGCTTTGGTC	AluSx
chr21_34774940	NA17773	GCTTTGGT/CGGC	13	3	CTTGTTAGCCAAGCTGGTCTCGAACTCCTGACCTCAGGTGATCCACCT	CTTTGGTCTCCCAAAGTGCTGGGATTATAGGCATGAGCCACCATGCCTGA	AluSx
chr21_34807620	NA17156	CT/TC	9	6	GATGACCATAACCTACCACAGACAGGGTCTCTCTCTCTCTCTCTCTCT	TTTTTCCCTGTTCTTGCGCCAGCTGCCTGACAATTTGTGGGATTAAAGCT	(TC)n
chr21_34807620	NA17460	TC/TC	15	13	GATGACCATAACCTACCACAGACAGGGTCTCTCTCTCTCTCTCTCTCT	TTTTTCCCTGTTCTTGCGCCAGCTGCCTGACAATTTGTGGGATTAAAGCT	(TC)n
chr21_34807620	NA17773	CTTTTTC/TCTTTT	12	8	GATGACCATAACCTACCACAGACAGGGTCTCTCTCTCTCTCTCTCTCT	TTTTTCCCTGTTCTTGCGCCAGCTGCCTGACAATTTGTGGGATTAAAGCT	(TC)n
chr21_34807776	NA17156	-/ATCC	21	6	TATTCTTTTCAGTGACTGCTCACTCCTCTTCCTCCAAAATCCCTATCC	TCCATCCATCCATCCATCCATCCATCCATCCATCCATCCATCCAACTTTC	(TCCA)n
chr3_38564678	NA17156	-/TTTTCCTACTCTCTTCTCCCCTCCT	42	31	CGATTAGTTCAGACAACATCTGGCACTTGATGTCTGTCCTTCCCTCCT	CTGGTCATTGTGCAGTTCTGGAAATTAAAAAGGTGACAGCCAGGCTAAAA	
chr3_38564674	NA17275	-/TCCTTTTTCCTACTCTCTTCTCCCC	52	38	TGCTCGATTAGTTCAGACAACATCTGGCACTTGATGTCTGTCCTTCCC	CCTGCTGGTCATTGTGCAGTTCTGGAAATTAAAAAGGTGACAGCCAGGCT	
chr3_38564678	NA17460	-/TTTTCCTACTCTCTTCTCCCCTCCT	60	42	CGATTAGTTCAGACAACATCTGGCACTTGATGTCTGTCCTTCCCTCCT	CTGGTCATTGTGCAGTTCTGGAAATTAAAAAGGTGACAGCCAGGCTAAAA	
chr3_38564678	NA17773	-/TTTTCCTACTCTCTTCTCCCCTCCT	52	39	CGATTAGTTCAGACAACATCTGGCACTTGATGTCTGTCCTTCCCTCCT	CTGGTCATTGTGCAGTTCTGGAAATTAAAAAGGTGACAGCCAGGCTAAAA	
chr3_38593427	NA17773	-/T	33	7	GAAGGGAGGCAGCATCTTCATGACGCACCCTGGGACCTGCACAGAGCC	CTTTTGCCTGTCAGGGCTGGGCCAAGGCTGGACCTCGGGGTTCCCGGGTC	
chr3_38596394	NA17156	CCCCACA/ACACACC	24	12	CACTCCCGGCTTGCGTGCGCGCACGCGCGCGCACACACACACACACAC	CCCACACACACCACACACACCCAGGAGCCAGAGTCAATGGGCTCAAGAGA	(CA)n
chr3_38596370	NA17773	GCGCGCGC/-	22	14	GACTTCCCTCATGAATCCCTGTGGCACTCCCGGCTTGCGTGCGCGCAC	CGCGCGCACACACACACACACACCCCCACACACACCACACACACCCAGGA	(CA)n
chr3_38596394	NA17773	C/ACA	26	13	CACTCCCGGCTTGCGTGCGCGCACGCGCGCGCACACACACACACACAC	CCCACACACACCACACACACCCAGGAGCCAGAGTCAATGGGCTCAAGAGA	(CA)n
chr3_38599800	NA17156	T/-	15	3	GGGAGGGGGCTGTGTGGGCTGGCATTAGCTCCCTCCTCCAGCAGCCTC	AATTTTCTCATCCCCAGGCAGATCTGCTGGGAAATCGCAGGGCTCCGTGT	
chr3_38602134	NA17156	GGTAGCTGGGTAGA/TGTGTGTGTGTGTG	13	5	GGTAGATGGATTGATAGAAGGGTAAGTAAATGGATAATTGGATGGGTG	GTAGCTGGGTAGATGAGTGGATGGTGTGTGTGTGGCCCTTGGCCAACTTA	(TGGA)n
chr3_38602154	NA17156	GATG/TGTGT	17	10	GGTAAGTAAATGGATAATTGGATGGGTGGGTAGCTGGGTAGATGAGTG	ATGGTGTGTGTGTGGCCCTTGGCCAACTTACCACAAGGTTGCCAATGACC	(TGGA)n
chr3_38602154	NA17460	GATG/TGTGT	22	9	GGTAAGTAAATGGATAATTGGATGGGTGGGTAGCTGGGTAGATGAGTG	ATGGTGTGTGTGTGGCCCTTGGCCAACTTACCACAAGGTTGCCAATGACC	(TGGA)n
chr3_38602134	NA17773	GGTAGCTGGGTAGA/TGTGTGTGTGTGTG	16	4	GGTAGATGGATTGATAGAAGGGTAAGTAAATGGATAATTGGATGGGTG	GTAGCTGGGTAGATGAGTGGATGGTGTGTGTGTGGCCCTTGGCCAACTTA	(TGGA)n

chr3_38602154	NA17773	GATG/TGTGT	18	11	GGTAAGTAAATGGATAATTGGATGGGTGGGTAGCTGGGTAGATGAGTG	ATGGTGTGTGTGTGGCCCTTGGCCAACTTACCACAAGGTTGCCAATGACC	(TGGA)n
chr3_38602661	NA17460	-/C	25	12	AGCTCCTGCCTGAAAGCCCAAGGAGCCCTTGGAGGGCACTGACACCTA	TCCCCCCCCGCCATTTCTCCCAGAGCACCCACTACTGTTTCTCTCCACAG	
chr3_38624928	NA17275	T/-	28	18	TGCCTCTGGCCTTCCTTCTGTTCCTCTGAAGACATCCTGCTGCCAGTG	CCCCATGCTCTCAGAAGACACGGGAAGCCCTGGGCAGGGCCTCAGCTCTC	
chr3_38624928	NA17773	T/-	33	16	TGCCTCTGGCCTTCCTTCTGTTCCTCTGAAGACATCCTGCTGCCAGTG	CCCCATGCTCTCAGAAGACACGGGAAGCCCTGGGCAGGGCCTCAGCTCTC	
chr3_38638341	NA17460	-/A	60	16	CAGAAGCAGTTTCTGGAACTGTGCAAATGGTTATAGGAGGCAGGACTT	GGAAAAAAAAGGCAGCAGAGTGTGTTACTGAAGTGGGGATAGCCCTGGGG	
chr3_38643195	NA17275	CACCC/GCCG	27	13	CTTGGGCTGCCTAAGTCTTCAGATCCCAGGGAGTTTAAAAAAAAAAAC	ACCCAAAGAAAAGGTAATTAACTCTGGAGGGGTATTTTCATGTGTTTGTG	
chr3_38643195	NA17773	CACCC/GCCG	31	13	CTTGGGCTGCCTAAGTCTTCAGATCCCAGGGAGTTTAAAAAAAAAAAC	ACCCAAAGAAAAGGTAATTAACTCTGGAGGGGTATTTTCATGTGTTTGTG	
chr7_150277455	NA17773	ACT/-	20	7	ACAGTAGTGAATCAAAACCAGCATTTTTTTTTTTTTTTTTTTTTTTTTT	CTGAAAGAACATACAGTAGTATAGCTTAGCACAGCACAGAACAGAAATGC	
chr7_150280333	NA17773	-/CTCTCTTT	30	10	TGCAGCTGCCTTGCCACCATGTCTCTCTCCCACTGTCTTTCTCTCTTT	TCTCTCTCTCTCTTTTTCTCTGTCCTCCTCGCCACCCCCTCCACCCCACT	(TC)n
chr7_150286415	NA17275	A/GAC	6	2	AGCGACTCGCTGCTGGGTGCCGCGGGCGTCAGGTCCACGTCCACCACC	CGGCCCCCGGGGCGCCCGCGCCGCCCGCGCCGCCCGACCGCACCGACGAC	GC_rich
chr7_150290675	NA17156	-/A	12	5	CTTACACACACACACGAGAGACAGAGAGAGAGAGAGAGAGAGAGAGAG	AGTGACTGCTGGCCCTCTTCCTTTGTCGAGATTCAAGAAATTTTTCCTTT	
chr7_150290653	NA17460	-/AG	13	3	CAGCACAAGCCTGTAACTCACACTTACACACACACACGAGAGACAGAG	GAGAGAGAGAGAGAGAGAGAGAGGAGTGACTGCTGGCCCTCTTCCTTTGTCG	(GA)n
chr7_150298286	NA17156	TC/-	5	2	CCACCCAGGAAGCCACCTCTGGGGAGAAAAGAGGCCCAGTCTCCCCCC	CACACACCCCCAAGCTGGCCCTTCTGCTGCCCCATGGCCCCCCTCCCCAG	
chr7_150298336	NA17275	-/C	5	2	ACACACCCCCAAGCTGGCCCTTCTGCTGCCCCATGGCCCCCCTCCCCA	AGCCCAAGGCCACCATCAAACTCTGCTCCCATCTCAGACAGCTCTTCGCA	
chr7_150298336	NA17460	-/C	3	2	ACACACCCCCAAGCTGGCCCTTCTGCTGCCCCATGGCCCCCCTCCCCA	AGCCCAAGGCCACCATCAAACTCTGCTCCCATCTCAGACAGCTCTTCGCA	
chr7_150299654	NA17275	-/A	26	14	GGGTGGGGGACAATGGGAGAGAGGGAGAGAGGCAAGGAGCAAGGTTCC	GGGTGCTGAGGCGAGGGAAGAAGACAGGAGGAGGCACCAGAGAGGAGATA	

Supplemental Table 15. Primer pairs used for long-range PCR amplification of the targeted interval (Perlegen Sciences mapped to NCBI build 36) and PCR amplification around discordant calls for validation by independent sequencing.

Application		Sequence	NCBI36 coordinates	Amplicon size (bp)
LR-PCR	LR-1527027_1	GAGGGGACAGAAGACCAGGCTGGCTAATG	chr7:150284619-150284648	
	LR-1527027_2	GGGCTGTCATCATGTTCATCCTCAATTTCGAG	chr7:150287673-150287705	3087
	LR-1977_1	GTAGTACCTTAGTACCCTTGCAGCCCGTTTC	chr21:34657259-34657290	
	LR-1977_2	CAAGATAGCAGGGCAGGGAATAAAGATCAGTA	chr21:34671453-34671485	14227
	LR-1983_1	TGTGTCTGAGGATTGTCTGGCGTGAGG	chr21:34735911-34735938	
	LR-1983_2	GCTCATTGGCATCTGTTCATTGGAGTAAGCAG	chr21:34746674-34746706	10796
	LR-1985_1	GGGACCCACATTGCTCTTAGAACTATTC	chr21:34760612-34760640	
	LR-1985_2	CTTGTAGCTGAACCCAACTGTATCACAACTTA	chr21:34775055-34775087	14476
	LR-1986_1	TGATACAGTTGGGTTCAGCTACAAGGGTAAC	chr21:34775063-34775094	
	LR-1986_2	CTTCTCTTTGCTAGTTGAACCCGCTACTACAT	chr21:34785499-34785531	10469
	LR-1987_1	CAACTAGCAAAGAGAAGACAGCGTCCTT	chr21:34785515-34785543	
	LR-1987_2	TATAGTGGATAACCTTGACTTACCCGGTCCTA	chr21:34795404-34795436	9922
	LR-1988_1	CAGAGAGTAGGACCGGGTAAGTCAAGGTTATC	chr21:34795398-34795430	
	LR-1988_2	CCACGCCAGGTCTTCTTTAGGATCTAGTCT	chr21:34799991-34800021	4624
	LR-1989_1	ATTACTGTAGCTCAAACGAGGCCAGACTAGAT	chr21:34799969-34800001	
	LR-1989_2	CGCATTTGACACTTGACTGGGATACACTA	chr21:34806349-34806378	6410
	LR-1990_1	CGGTAGTGTATCCCAGTCAAGTGTCAAA	chr21:34806347-34806375	
	LR-1990_2	CCGTAAGTAGTGTCTATGTATTGGCAACTGTT	chr21:34818417-34818449	12103
	LR-240580_1	GGGCCAGGCTAGGTTTGCTATTGCGATGTCAG	chr11:73836950-73836982	
	LR-240580_2	GATCTAACACGAGAGGGCTGGGACGGTGGA	chr11:73845920-73845950	9001
	LR-240581_1	CCCAGCCCTCTCGTGTTAGATCATAGACA	chr11:73845929-73845958	
	LR-240581_2	CACAGAGCCAAGCATACAGCCAAGATTAGAAT	chr11:73854354-73854386	8458
	LR-240582_1	CTTTCATGATTCTAATCTTGGCTGTATGCTTG	chr11:73854347-73854379	
	LR-240582_2	CATCCTCCACCCACATTCCTTAGTTCT	chr11:73862538-73862565	8219
	LR-382423_1	ATAGAGATTCCTCATAGTCTTGGGCTACACTG	chr2:223616214-223616246	
	LR-382423_2	CCTTCTAGGTCTCAGCTACGTGACGAAAT	chr2:223627188-223627217	11004
	LR-54275_1	CCAAGGATGAGGGAGTCTGCGTCACAAG	chr7:150269610-150269638	
	LR-54275_2	TGGTCATCTACACGGCTGTCTTCACACCCTAC	chr7:150280720-150280752	11143
	LR-54276_1	GCAGCCGAGTAGGGTGTGAAGACAGCCGTGTA	chr7:150280713-150280745	
	LR-54276_2	CTGTGCAGGGCCTGGTCTCCACTCTCGATCTA	chr7:150285585-150285617	4905
	LR-54277_1	TCTAGAATCCAGTCATCATAAACCGCCATA	chr7:150286689-150286719	
	LR-54277_2	AAACTCCTCCCTTAGAGAACTTCTGCGTTTAG	chr7:150298143-150298175	11487
	LR-54278_1	GAGTTTCAGGAACGCTTCAGGGAAATACCAAC	chr7:150298170-150298202	
	LR-54278_2	GTTCGCTCACAGCAAATCAGGGTTCTAACTG	chr7:150301561-150301592	3423
	LR-721496_1	CTGGACGTGTCATGTAAATTCTTGATTGGTTA	chr3:38553978-38554010	
	LR-721496_2	ACCCATGCCCCTGACAGATCTAGTTCT	chr3:38565051-38565078	11101
	LR-721498_1	TGACATTCTGAGGGACAATTGCAGGAATAGAC	chr3:38575485-38575517	
	LR-721498_2	GAGCCCTAGTGACGTGTTCAGTTGCCTAGA	chr3:38584174-38584204	8720
	LR-721499_1	CACCTCCTCATTTCTGCCTCAACTCAA	chr3:38584039-38584066	
	LR-721499_2	CAAACTCTGATGCAGGACTAACCCACTACCTC	chr3:38595574-38595606	11568
	LR-721500_1	CTCAGGGGTTGCGAACTCCAATGCTTAC	chr3:38595323-38595351	
	LR-721500_2	TTCCTGAGTCCTAGCTCCACCTGATAACTGCC	chr3:38606373-38606405	11083
	LR-721501_1	CAGCTGTTTTGGTGGTGAGATGGCAGTTATC	chr3:38606353-38606384	
	LR-721501_2	CCAGATTGGGAGGTAGGGGCTGAAGACTTACT	chr3:38618289-38618321	11969
	LR-721502_1	CCCAACCCTGTGCTTTGTTTTCAGACTAGAAC	chr3:38618395-38618427	
	LR-721502_2	CATTGTCTCGTGTGTGAATTTCCCTTGTTACA	chr3:38630329-38630361	11967

	LR-721503_1	TGTAACAAGGGAAATTCACACACGAGACA	chr3:38630330-38630359	11821
	LR-721503_2	TAACAAATAATTCCCTCCCTCCTGTACGGTAG	chr3:38642118-38642150	
	LR-721504_1	ACCGTCGAATGTGGGGAGACTACCGTA	chr3:38642100-38642127	11891
	LR-721504_2	TCAGGGCACACAGAATTTTGGAGAATGTCTAA	chr3:38653958-38653990	
	LR-721505_1	ATTGTAAGACCACAGTCGCTCATCCCCTAGAT	chr3:38653478-38653510	11811
	LR-721505_2	GGCTCTCGACCTCTGTGTTATTTGATCGTC	chr3:38665258-38665288	
	LR-778623_1	ATGGATACAGGCCCTAAATTTGAGAGTCTAT	chr21:34746238-34746269	3341
	LR-778623_2	AGGATGATTTCTGATGGCATATTTAGGTAAGA	chr21:34749546-34749578	
	LR-778624_1	CAGCTGAGGAATAGGCCACTTTTATCAGATAG	chr21:34749486-34749518	11646
	LR-778624_2	ACTTATTCCTCCTGCTTACCTTCCTAGTCCAA	chr21:34761099-34761131	
False positive Validation	FP1-F	CAGTGTTCCTACCCCCTCTG	chr21:34759544+34759840	297
	FP1-R	CTGGTTCTCTGGCTGGGTAA		
	FP2-F	GCTCACTCCTCTTCCTCCAA	chr21:34807745+34808040	296
	FP2-R	GAATCGTTGCCAGCTACTCC		
	FP3-F	TCAATGGGCTCAAGAGAAGG	chr3:38596428+38596733	306
	FP3-R	GTTGAGTCAGGCCCATCTTG		
	FP4-R	GAGGATGGTGCACATGATGA	chr3:38638951+38639257	307
	FP4-F	CCCTACCAGCAGTGTGGTTT		
	FP5-F	GGCTGTGCAGTGTCAGGTTA	chr7:150292849+150293143	287
	FP5-R	GGAGGCAGGAGCTTAGGACT		
	FP67-F	GAGTTAGGGAGCCCAAGAGG	chr7:150293004+150293290	271
	FP67-R	CCCCCAAGACCCTATAAATG		
False Negative Validation	FN1-F	AGGCAGAAGACAAAGGCAAG	chr7:150299734+150300033	300
	FN1-R	GCAGAGTCCAGTGGTGTGAG		
	FN2-F	GGGCTAGGAATGGAAGAAGG	chr7:150275218+150275518	301
	FN2-R	TCCAACATTTTCAGCTTCTGG		
	FN3-F	GGAACAAAACCTGCCCATA	chr2:223624363+223624672	310
	FN3-R	TTAACCTCTCCGCACCTCAC		
