# Supplementary material for: Evaluation of next generation sequencing platforms for population targeted sequencing studies
Source: Genome Biol. 2009 Mar 27;10(3):R32. doi: 10.1186/gb-2009-10-3-r32 (PMC2691003; doi:10.1186/gb-2009-10-3-r32)
Supplement: Additional data file 2 — Additional information to the methods used and calculations performed for this study. [file gb-2009-10-3-r32-S2.doc]

Supplemental Methods

**Long-range PCR amplification**

***Sample preparation***

28 LR-PCR reactions were performed to amplify six genomic intervals spanning a total of 266-kb in each of four DNA samples (NA17275, NA17460, NA17156, and NA17773) obtained from the Coriell Institute (www.coriell.org/). We performed the 28 LR-PCR reactions using 30 ng of genomic DNA, 0.5 µM forward LR-PCR primers, 0.5 µM reverse LR-PCR primers (Supplemental Table 15) in a total reaction volume of 12 µl, as described^1^. Following LR-PCR, the 28 amplicons generated using a single DNA sample template were quantified and combined in equimolar amounts. We attempted 28 LR-PCR reactions in each of four DNA samples (NA17275, NA17460, NA17156, and NA17773), and only two reactions failed coming from two different samples (NA17156 and NA17275). Following LR-PCR, the 28 amplicons generated using a single DNA sample template, ranging in size from 3,088 bp to 14,477 bp, were quantified by the PicoGreen dye binding assay, combined in equimolar amounts, and used to create libraries for Roche 454, Illumina GA and ABI SOLiD sequencing.

***Reference Sequence***

The reference sequence used for aligning sequence reads generated by all three NGS platforms was constructed by extracting the following positions from the UCSC Genome Bioinformatics site (genome.ucsc.edu) build hg18 (NCBI build 36): chr11:73836950-73862566; chr21:34656259-34672486; chr21:34734911-34819450; chr2:223615214-223628218; chr3:38553978-38665289; and chr7:150268610-150312098.

***Fold-enrichment of targeted sequences***

We determined how many of the generated Roche 454 reads map to the targeted intervals versus other locations in the genome. We chose to perform this analysis with the Roche 454 reads due to them being, on average, 10 fold longer than the Illumina GA or ABI SOLiD and therefore less prone to alignment difficulties. Of the 197,461 sequence reads generated from the four DNA samples 72% mapped uniquely to the reference genome. Unique BLAST alignments were defined as those that at least 25 bp and 80% sequence identity, subsequent analysis determined these alignments were typically full length and had on average more than 96% sequence identity. Of the 72% of reads with unique alignments in the genome most of them (97.8%) align to sequences in the 266-kb targeted intervals. Since we are aligning to the whole genome for this analysis rather than to the 266 kb reference sequence, a lower percentage of reads than what is indicated in Supplemental Table 1 are uniquely aligned.

We sought to determine the enrichment of targeted genomic loci using LR-PCR amplification. An enrichment of >4,300 fold is calculated as follows: genome/(target+other non-redundant non-target sequence) having a maximum theoretical enrichment of >11,500 (genome/target - 3,000,000,00/260,000). In addition to this analysis, the large fraction of Roche 454 reads that align to the targeted sequence (Supplemental Table 1) provides further evidence that the long range PCR sample preparation method amplifies the targeted sequences with high specificity. The LR-PCR fold enrichment obtained in this study compares very favorably to 432 fold enrichment measured from microarray based capture methods followed by Roche 454 sequencing^2^.

**Generation and analysis of Roche 454 sequence reads**

The laboratory methods and protocols used in this experiment were as described by Rothberg and coworkers^4^. For each of the four samples consisting of 28 equimolar pooled PCR products, 3 μg of DNA was used for the library preparation step, following the standard protocol for whole genome libraries. Each pooled sample was sequenced on one region of a four region plate using 300,000 enriched template beads per region, following Roche 454 guidelines.

The reads produced by the Roche 454 FLX platform were mapped to the reference sequence using two alignment algorithms, Newbler (provided by Roche), and BLAST. Valid alignments produced using the default parameters of Newbler included both full and partial read alignments with the vast majority being unique alignments on the local reference sequence. Unique BLAST alignments were identified that had at least 25 bp and 80% sequence identity and subsequently analysis determined these alignments were typically full length and had an on average more than 96% sequence identity. Due to the uniqueness criterion, the BLAST alignments were more conservative than the alignments produced by Newbler (see Supplemental Table 1) where on average 82% of reads aligned (BLAST) compared to 95.5% of reads (Newbler). Unless specifically noted all analyses reported in this study used the reads aligned to the reference sequence using the Newbler algorithm.

DNA variations in the read sequences were identified directly from the alignments of multiple reads either by directly accessing such variation in the AllDiffs file produced by Newbler or by custom programs working on BLAST output. Sequence reads aligned by both methodologies were analyzed for variants. To call a heterozygous or homozygous alternate base both the absolute number and frequency of reads considered (Supplementary Figure 1A). To call a variant: 1. at least two reads had to contain the alternate base and 2. A heterozygous variant was call if 20%-80% of the reads contained the alternate base and a homozygous variant was called if greater than 80% of the reads contained the alternate allele.

Roche 454 identified 242 indels in the 260-kb of analyzed sequence. Many of which are located in homopolymer stretches or simple/low-complexity repeats sequence contexts where indels are more likely to be present. Further analyses are required to validate these indels as true variants.

**Generation and analysis of Illumina GA sequence reads**

The Illumina GA libraries were prepared and sequenced according to the manufacturer’s instructions using the provided kits (version 1) and starting from the 28 equimolar pooled PCR products, except for the fragmentation step which was performed as follows: 1 µg of DNA was incubated for 25 min at 37ºC with 0.25 U of DNase I resulting in digestion to the 170 to 250 bp fragment size range. Cluster densities ranged from 17,700 to 22,300 per tile for each of the four samples. We used the Genome Analyzer Pipeline 0.2 with default signal quality filters (chastity of base signals >0.6 within the first 12 bases of the read) in order to qualify passing reads.

We compared two alignment tools, ELAND 0.2 (Efficient Large-Scale Alignment of Nucleotide Databases), provided by Illumina, and MAQ 0.6.2.^5^. For the 4 individual samples, we aligned the ~3.2 million sequence reads that passed quality filters, using both ELAND and MAQ set to their default parameters. While ELAND aligned the first 32 nucleotides of each read with 2 mismatches allowed, MAQ aligned the entire 36 nucleotides read with a maximum of 2 mismatches in the first 24 nucleotides and maximum sum of mismatching bases quality score of 70. This alignment strategy, although taking advantage of the quality scoring of the bases, seems more stringent than ELAND simple mismatch evaluation as it aligns slightly fewer reads (Supplemental Table 1). To maximize the quality of the Illumina GA sequence data, we implemented a post-processing quality filter for the alignments generated by both the ELAND and MAQ algorithms. This conservative filter looks at the alignments, and counts the call for each base only if it reaches the required threshold (e.g. Bustard Q-score=30 for ELAND or Q-score=20 for MAQ calculated as ASCII_maq_-33), regardless of its position in the read.

We analyzed the Illumina GA sequence for variants using the alignments generated by both ELAND and MAQ. Using the same criteria as that applied to the Roche 454 sequence reads, to call a heterozygous or homozygous alternate base both the absolute number and frequency of reads were considered (Supplemental Figure 1B). To call a variant: 1. at least two reads had to cover the base and 2. A heterozygous variant was called if 20%-80% of the reads contained the alternate base and a homozygous variant was called if greater than 80% of the reads contained the alternate allele. When comparing the Illumina GA-generated sequence data to that of the Sanger sequence data generated with the ABI 3730XL instrument, the post-processed, quality filtered Illumina GA reads aligned by both tools gave very similar results for all the metrics measured (accuracy of base calls, sensitivity to detect variants, false positive rates). An important advantage of MAQ is the flexibility with which it generated the alignments to the 266-kb reference sequences, thus allowing for more rapid assessment of different filters and parameters. This is the reason why we have selected it for use in all the analyses we report in this study.

ABI SOLiD sequence reads

***Library Preparation, Emulsion PCR, Enrichment and Sequencing***

Short fragment DNA libraries were generated from the four 28 equimolar pooled amplicon samples and sequenced using standard ABI SOLiD protocols at Applied Biosystems in Beverly, MA. Briefly, the short fragment DNA libraries first underwent titration emulsion PCR followed by sequencing to determine the optimal template concentration. After this, the libraries were clonally amplified on beads using large-scale emulsion PCR. The emulsion PCRs were then broken and washed to purify the emulsified beads, enriched for templated beads, and 3’-end modified to allow the beads to attach to the sequencing slide. Each library was run on a single slide using the 25bp fragment run module. There was a fluidics issue with sample NA17460 that resulted in bead loss.

***SOLiD Data Analysis***

Applied Biosystems provided the ABI SOLiD sequence data in a tab-delimited file. For each sample, ABI had aligned the sequence reads to the reference sequence. As output, they provided at each position, the number of valid and invalid nucleotide observations. We defined total raw coverage as the total number of observations at a position (both valid and invalid) and high-quality coverage as the number of valid nucleotide observations at a position (A or C or G or T).

We created our own parameters for calling variants from these data files so that we would be able to perform simulation analyses. We used both ABI SOLiD variant calling software and comparison to known Sanger genotypes (Supplemental Figure 1C) to establish these parameters. Our parameters performed similar to or better than the calls by ABI SOLiD software; averaged over the four samples, the variant accuracy exceeded ABI SOLiD’s by 1% based on the comparison with Sanger sequence data. The parameters for calling variants are as follows, where N refers to the number of invalids:

1. Homozygous reference: This follows ABI SOLiD’s calling parameters. If the frequency of the reference base: # reference bases / (A+C+G+T+N) exceeds 0.25 and all other base frequencies are less than 0.1, this is a homozygous reference
2. Heterozygote: If Ref / (A+C+G+T) > 0.2 and Alt / (A+C+G+T) > 0.2 and there are a minimum of 2 reads for both the reference and alternate allele and the top 2 bases are the predominant calls (Ref + Alt) / (A + C + G + T + N) > 0.75. This differs from ABI SOLID’s algorithm for calling heterozygotes which tests multiple conditions for calling a heterozygote. In ABI SOLiD’s software: Alt/(A+C+G+T+N )> 0.25 OR (Alt /(A+C+G+T+N) > 0.2 AND Alt/A+C+G+T) > 0.4). For the reference allele: Ref / (A+C+G+T+N) > 0.25 OR (Ref / (A+C+G+T+N) > 0.2 AND Ref/(A+C+G+T) > 0.4)
3. Homozygote: This closely resembles ABI SOLiD’s calling parameters. If Alt/(A+C+G+T+N) > 0.5 OR (Alt/(A+C+G+T+N > 0.3) AND Alt /(A+C+G+T+N) > 0.5), and there are 2 calls supporting the homozygote, the homozygote is called. ABI’s SOLID’s SNP calling algorithm requires at least 2 unique start points, rather than 2 calls. For the simulation analyses we were unable to simulate unique start points, and therefore changed the restriction to 2 reads.

We noted that when we graphed the alternate allele frequencies (Supplementary Figure 1C), the distribution for heterozygous loci is shifted to the left and centered at 0.42 instead of 0.5. This was unexpected and unlike the distributions observed for Roche 454 and Illumina GA. This may be due to SOLiD’s alignment method. ABI SOLiD sequences nucleotide bases in color space where variants count as 2 colors mismatches. Because ABI SOLiD reads are only aligned up to 4 color mismatches, some variants are lost due to a bias for aligning to the reference sequence. The significance of this is that there is a slight bias against calling variants that differ from the reference sequence.

**Data from short range PCR and Sanger sequencing**

We employed an existing data set deposited by JCVI into dbSNP (submitter handle: RSG_JCVI) performed under the auspices of the National Heart, Lung and Blood Re-sequencing and Genotyping program (<http://rsng.nhlbi.nih.gov/>). The data used in this study represents a small portion of the dbSNP data set, 4 Coriell DNA samples out of a total of 188, produced for a project by Dr. Dan Roden who provided permission to use supporting Sanger trace data and the details of the regions scanned. In brief, the data set included 88-kb of non-contiguous sequence encompassing the exons and the intronic sequence conserved with mouse and rat in the K^+^/Na^+^ channel proteins produced by employing 273 short-range PCR reactions generating amplicons averaging 418 bp in length. The methods employed for performing short range PCR amplification and capillary electrophoresis using Sanger chemistry are available at <http://www.ncbi.nlm.nih.gov/SNP/snp_viewTable.cgi?method_id=4738>.

We inspected ABI Sanger sequence traces at loci for which all three NGS technologies were concordant in their calling of a base as either reference or variant but discordant with the ABI Sanger genotypes to determine the quality of the electrophoregrams. Trace inspection enabled us to explain discordant NGS genotypes (either identified as false positive, false negatives or discrepant variants) in relation to the corresponding ABI Sanger genotype. Occasionally, variant calls were missed due to adjacent heterozygous indels variants which results in mixed peak occurring downstream of an indel. Digital signal processing algorithms were developed for ABI Sanger traces to improve detection of long and short alleles comprising the indel but we found that occasionally these filtering methods did not adequately separate the allelic forms. Also, ABI Sanger trace peak anomalies are caused due to the presence of mono- or di-nucleotide repeats which cause PCR to stutter.

**Overrepresentation of amplicon end sequences**

Amplicon end sequences compose about 2.3% (~ 6 kb) of the targeted intervals but account for up to 56% of the sequenced base pairs for Illumina GA technology. This extreme sequence coverage bias results from overrepresentation of the 28 amplicons ends in the DNA samples after fragmentation prior to library generation. Fragmentation of a ~10 kb amplicon down to a size of ~200 bp results in internal base pairs becoming end sequences which are used as the start site for sequencing. The base pairs at the amplicon ends are always end sequences after fragmentation, resulting in them frequently being used as start sites for sequencing.

For the ABI SOLiD platform, an amplicon end depletion protocol was employed as described in ABI SOLiD protocols; briefly the pooled PCR products were end labeled with biotin, fragmented, and then a streptavidin bead-based pull out method resulted in a depletion of the PCR product ends. This method successfully reduced the PCR product ends in the samples with NA17156, NA17275, NA17460, and NA17773 having 8.7%, 8.2%, 10.7%, and 9.7% of the sequenced base pairs composed of amplicon ends, respectively.

Compared to Illumina GA and ABI SOLiD the Roche 454 technology fragments the DNA to a larger size (~ 600 bp) prior to library generation and sequences less DNA molecules. These process differences results in less imbalanced representation of amplicon end bases, and the amplicon ends compose only 5% of the total sequenced bases.

**Source and analysis of sequence depth coverage variation**

***Sample to sample variation***

All three NGS platforms show variability in the average sequence coverage depth of the analyzed base pairs for the four samples. While we attempted to load equivalent amounts of each sample the average sequence coverage for Roche 454 ranges from 28x to 61x, for Illumina GA from 130x to 263x, and for ABI SOLiD from 537x to 1121x (Supplemental Table 2). Since the relative sequence coverage for a given sample on one NGS platform was unrelated to its coverage on the other two platforms, these observed differences likely result from variability in sample processing on each NGS platform rather than differences in the starting material of the four samples. NGS experimental designs need to take this sample to sample variability into consideration to ensure sufficient coverage is obtained for all samples in the study.

***Analysis of sequence coverage of repetitive elements***

For the entire 260-kb of analyzed sequence the average and standard deviation of coverage depth was calculated for different types of repetitive elements (RepeatMask: Alu, LINE, SINE, Simple repeats, Low complexity repeats) defined by their annotation and genomic coordinates obtained from the UCSC genome Browser (hg18). Unique sequences correspond to the complement of the RepeatMask sequences. Relative depths of coverage were calculated as the ratio of the average coverage depth for a given type of repetitive element to the average coverage depth for unique sequences in the same sample (Supplemental Table 4). For the NGS platforms, the coefficient of variance was calculated for each sample as the ratio of the standard deviation to the mean coverage depth for the region of interest. For each amplicon, we took the mean and standard deviation of coverage depth for that amplicon, and calculated the coefficient of variance. The coefficient of variance was then averaged over all 28 LR-PCRs. This same analysis was carried out again, but restricting to the unique non-repetitive sequence in each amplicon.

***Analysis of coverage variability between amplicons***

For the three NGS platforms, coverage variability between amplicons for each sample was calculated as follows: the average coverage of unique sequences (non-repeatmasked) was calculated for each amplicon; this coverage was then normalized to the median coverage of all the amplicons; the distribution of this normalized coverage as well as the fold difference in coverage within 90% of the amplicons was determined (Supplemental Table 3).

**Regions of Low Coverage**

For each of the three NGS technologies we examined the sequence composition of base pairs with low coverage. To identify trends we required that at least 2 of the samples have low coverage defined as positions with approximately less than 5% of the average depth of coverage. The coverage of Roche 454 averaged over all four individuals is 43, hence for Roche 454, we examine positions with two or less reads (5% of 43). The average coverage of Illumina GA across all four individuals is 138, hence we examined positions with nine reads or less (5% of 138). The average coverage for ABI SOLiD is 840, hence we examine positions with forty-two reads or less. For each technology, low-coverage positions within 5 bp of each other were combined into a single low-coverage interval.

For the Roche 454 sequence data there was a single no-coverage interval spanning 59 bp and another 4 bp low-coverage interval (Supplemental Tables 5 & 6). Inspection of the raw 454 read alignments in the locale of this 59 bp no-coverage interval in sample NA17773, revealed reads with low quality bases spanning this region and that read assembly generated the correct consensus sequence. For the Illumina GA sequence data there were two no-coverage and thirteen low-coverage intervals, which spanned a total of 272 bp, were predominantly repetitive, and the majority of the intervals had high AT-content (on average, 82%). For the ABI SOLiD sequence data there are 40 no-coverage intervals totaling 464 bp and 168 low-coverage intervals totaling 3,415 bp, most of which tended to be AT-rich (on average, 85%).

**Allelic Imbalanced Amplification**

Out of the 28 LR-PCR amplicons in the study there are 23 amplicons with heterozygous positions in at least one of the four samples which were identified in the Sanger sequence data (Supplemental Table 7). For four of these amplicons, skewed alternate allele read frequencies (AARFs) at the heterozygous positions likely reflect an allelic imbalanced amplification in one or more of the four samples. For example, the LR-PCR amplicon that spans chr21:34657259-34671486 demonstrated severe allelic imbalanced amplification for all working samples where the vast majority of heterozygous positions in the Sanger 3730xL data set were called homozygous for either the reference or alternate base. On the other hand, the amplicon spanning chr11:73836950-73845951 showed moderate allelic imbalanced amplification in two of the four samples where ~80% of the heterozygous positions were correctly called but their AARF were shifted off center. The other two affected amplicons show allelic imbalanced amplification in only one of the samples with heterozygous SNPs in the interval.

***Detecting Allelic Imbalance***

The AARFs for Sanger-identified heterozygotes have the following distribution for each of the three technologies:

Illumina: μ _Illumina_ = 0.49, σ^2^ _Illumina_ = 0.04

454: μ_454_ = 0.48, σ^2^ _454_ = 0.05

SOLiD: μ_SOLiD_ = 0.42, σ^2^ _SOLiD_ = 0.04

A SNP has 3 AARFs, one from each NGS technology. Then the weighted average will be normally distributed with mean = (μ _Illumina_ + μ_454_ + μ_SOLiD_) / 3 and variance = 1/9 * (σ^2^ _Illumina_ + σ^2^ _454_ + σ^2^ _SOLiD_ ) or mean = 0.46, variance = 0.015

If there are *n* SNPs in an amplicon, and it is examined by all 3 technologies, then the average AARFs of the *n* SNPs is 0.46 with variance (1/*n*^2^)*0.015. We performed a one-sample z-test comparing the observed AARFs to the expected mean. Amplicons with p-values < 0.01 are highlighted in gray in Supplemental Table 7.

For future projects increasing the number of amplicons such that each base is covered by at least two would efficiently reduce this bias and give more accurate variant base calls^3^.

**Simulations**

Simulations were performed in order to assess performance of each NGS platform at lower coverage depths. We used two different simulation approaches, which were tailored to the raw data available from each NGS technology, to produce lower coverage data sets. The first simulation approach (method 1) randomly sampled a subset of the reads and recalled genotypes. This was used to assess the Roche 454 and Illumina GA technologies. The second simulation approach (method 2) randomly sampled positions and the bases called at each position, independently of each other, given known prior probabilities. We used method 2 for ABI SOLiD simulations since the reads were not available to apply method 1.

***Simulation method 1***

In order to simulate different levels of read depth coverage for Roche 454 and Illumina GA, distinct percentages of reads were employed for each donor such that a defined level of coverage was obtained for all four donors. Ten randomly selected read subsets (independent trials) were generated per donor per defined level of coverage. For Roche 454 sample NA17275 had an average sequence coverage of 28x (Supplemental Table 2) and was not simulated at depths higher than what was experimentally observed. For Roche 454, the placement of a read was obtained from the original analysis and for Illumina GA, the reads were realigned to the reference sequence, using the parameters previously described. Genotypes were called using the reads from the randomly selected subsets with the same variant calling algorithms as described previously. For each trial, a set of performance metrics was obtained by comparing the resulting simulated genotypes with ABI Sanger sequencing genotypes.

***Simulation method 2***

In order to simulate performance at lower coverage for ABI SOLiD technologies, we simulated the coverage at each sequenced position, and then simulated what DNA bases would be observed at that position, conditional on the number of reads. The simulations used probabilities from the original coverage and read files that had on average ~840 fold-coverage for ABI SOLiD.

The simulation was done in two steps. The first was to simulate the depth coverage at each position. The purpose for this is demonstrated in the following example. When sequencing at 10x, not every position will have 10 reads. Some positions will have fewer reads, and some positions will have more reads. Technology which sequences exactly 10 reads for 100% of the positions is more valuable than technology that sequences 0 reads for 50% of the positions and 20 reads for the rest of the 50% positions, even though both have an average 10x coverage because the latter will be unable to call variants in half of the positions. Thus it is important to capture the variability in coverage across a sequenced region.

To target different levels of coverage, we took the total number of base pairs that were sequenced for each individual, *amp_seq*. We multiplied this by the desired coverage *cov*. Then the total number of base pairs that are sequenced at coverage *cov* is *tot_bp* = *amp_seq* * *cov*. *Tot_bp* is distributed among the sequenced amplicons. For each position in the amplicon, we assign the probability that a read lands there is proportional to the coverage observed in the real data files. From the original coverage file, we calculate the fraction of reads that are attributable to position, *pread_pos*. Therefore, a position that has 500 reads in the original data is 5X more likely to be selected to have a read than a position with 100 reads. For *tot_bp* events, we select a position based on *pread_pos*. Therefore, positions that had high coverage in the original file are likely to have higher coverage in the simulated file and positions with low coverage in the original file are likely to have lower coverage. Thus, we generate the number of reads at every sequenced position.

Given the number of reads at a position, we next simulate what DNA bases are called at that position. Again, we go back to the original data file and calculate the frequencies for the bases that are called at that position : f_A-pos_, f_C-pos_, f_G-pos_, f_T-pos_, f_N-pos_. Besides the four valid nucleotide bases (A,C,G,T), we also include invalid or low quality bases “N”. For ABI SOLiD, N-pos is the fraction of invalids or errors. It is important to include N’s (errors, invalids, and/or low-quality calls) in the frequencies, because if one just considered the valid nucleotides, then every base that is sequenced would contribute the genotype call. f_N_ is currently very high, on average 9%. This effectively reduces the sequence coverage because these low-quality/erroneous calls are thrown out. To simulate base calls at a position, we draw from a multinomial distribution, where the total number of trials is equal to the coverage at that position, and the aforementioned frequencies to call the bases. The output of each simulation consists of the read coverage and the types of bases observed at that position. This dataset is processed by the variant calling algorithms, and genotypes are called and compared to the ABI Sanger genotypes.

**Alignable Sequence versus Raw Sequence Reads**

We note that the coverage rate in the simulations (Figure 5) refers to alignable sequence. For Illumina GA and ABI SOLiD, ~43% and ~34% of the raw sequence reads are alignable on average, respectively (Supplemental Table 1). The fraction of alignable reads must be taken into account when designing experiments. For example, if one wanted to sequence 100 kb at 50x coverage, this would be equivalent to sequencing 5 Mb. Then to reach 50x of alignable coverage, one needs to generate 11.6 Mb (5 Mb / 0.43) of raw bases with Illumina GA. Similarly, on the ABI SOLiD, one would need to generate 14.7 Mb (5 Mb/0.34) of raw bases. The Roche 454 platform exhibits better than 95% of alignable reads (Supplemental Table 1) therefore, raw sequence and alignable coverage are more closely related and 5.3 Mb (5 Mb/0.95) sequenced bases is sufficient to achieve the desired fold coverage. Effectively, a 50x aligned coverage implies ~53x raw bases sequenced by Roche 454, ~120x raw bases sequenced for Illumina GA, and ~150x raw bases sequenced for ABI SOLiD. Therefore, when designing experiments and calculating the target coverage for a region, one must consider the fraction of alignable sequence.

References

1. K. A. Frazer, E. Eskin, H. M. Kang et al., *Nature* **448** (7157), 1050 (2007).

2. T. J. Albert, M. N. Molla, D. M. Muzny et al., *Nat Methods* **4** (11), 903 (2007).

3. A. R. Quinlan and G. T. Marth, *Nat Methods* **4** (3), 192 (2007).

4. M. Margulies, M. Egholm, W. E. Altman et al., *Nature* **437** (7057), 376 (2005).

5. H. Li, J. Ruan, and R. Durbin, *Genome Res* (2008).

6. Laura Bonetta, *Nat Meth* **3** (2), 141 (2006).
