# Supplementary material for: Evaluation of next generation sequencing platforms for population targeted sequencing studies
Source: Genome Biol. 2009 Mar 27;10(3):R32. doi: 10.1186/gb-2009-10-3-r32 (PMC2691003; doi:10.1186/gb-2009-10-3-r32)
Supplement: Additional data file 3 — Supplemental Figure 1: coverage distribution as a function of GC content for the 3 NGS. Supplemental Figure 2: distribution of alternate allele read frequency obtained for each NGS. Supplemental Figure 3: Sanger Traces of eight loci found to have discrepant calls between Sanger and NGS. [file gb-2009-10-3-r32-S3.pdf]

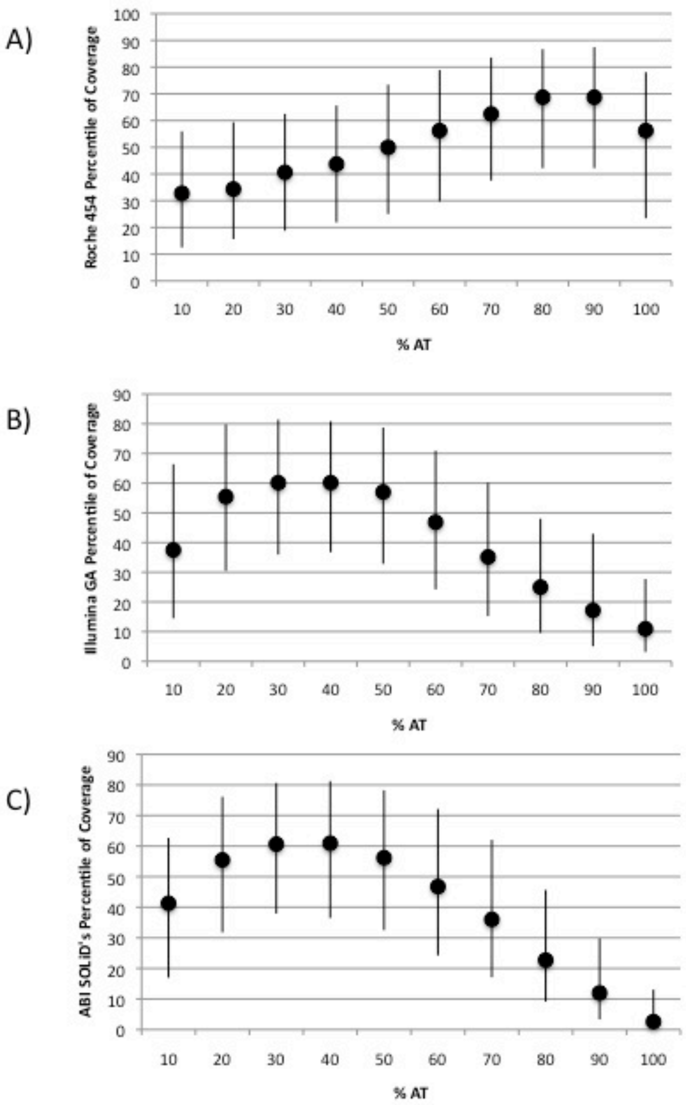

Supplemental Figure 1. **Read depth coverage from short-read NGS technologies decreases with increasing AT content.** For each NGS technology, nonoverlapping 10-bp windows were scored for their AT content and their percentile coverage relative to the entire sample. All windows with a particular AT % content were grouped together (x-axis). Then for each group, the median and the quartiles of the percentile coverage was plotted. If there are no sequence effects, then one should observe that the median percentile coverage, regardless of AT content, to be the 50th percentile. For Illumina GA (B) and ABI SOLiD (C), the median percentile coverage decreases as AT content increases. Roche 454 (A) does not have this trend.

For example, to interpret the 10-bp windows with 100% AT content for Illumina GA, look at the point on the bottom right of the figure B. The median coverage of the 100% AT windows is the 11th percentile relative to all other windows. The lower and upper-quartile of these 100% AT 10-bp windows have 3-28th percentile of coverage, indicating that AT-rich sequence tends to have lower coverage.

For simplicity, the percentiles of coverage from all four individuals were combined, and 0-10% AT windows were combined because there were not enough points at 0% AT.

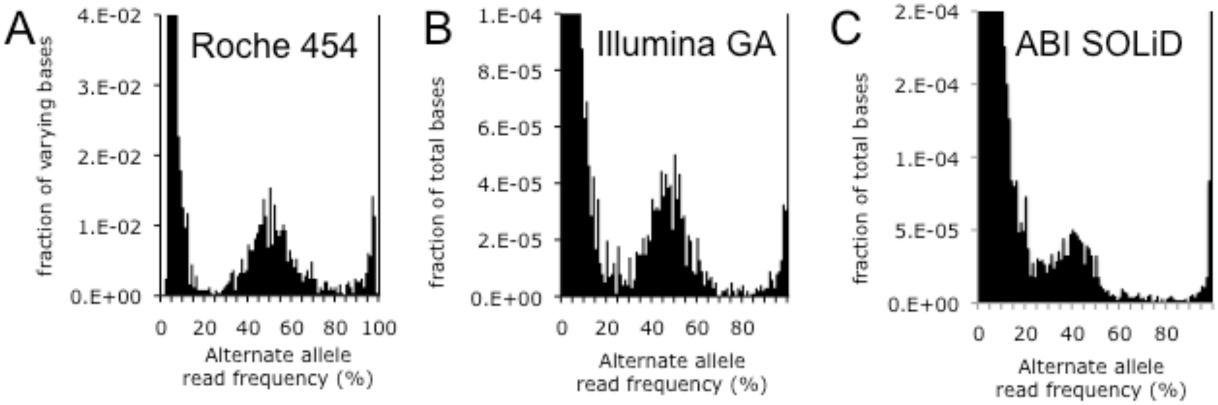

Supplementary Figure 2. We used the alternate allele read frequency distribution as the basis for establishing thresholds for calling variants. We define the alternate allele as the most commonly called base (which is not the reference base) for a given position in the reference sequence. Then the "alternate allele read frequency", or AARF, is the fraction of reads corresponding to the alternate allele. The fraction of the varying (A: Roche 454) or total (B: Illumina GA; C: ABI SOLiD) number of bases (y-axis) is plotted for every position.

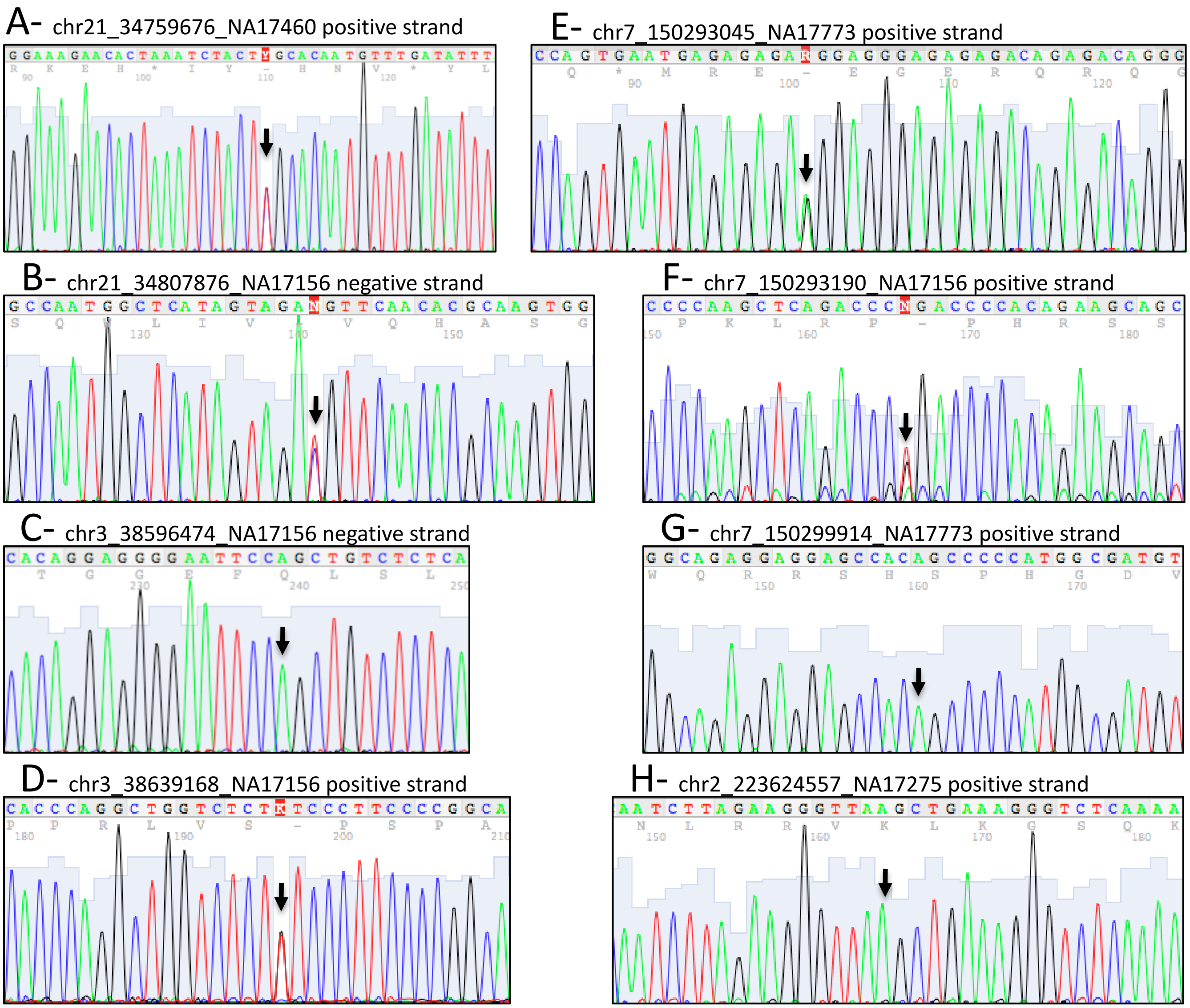

Supplemental Figure 3 : Validation of variant calls discordant between ABI Sanger and the 3 NGS platforms. Eight out of ten discordant variants calls were successfully amplified by PCR and subject to Big-Dye terminator sequencing. **(A-F)** Sequencing traces of PCR products of 6 False Positive calls (variant in 3 NGS and reference in ABI Sanger). Only **(C)** chr3_38596474_NA17156 is consistent with the original ABI Sanger call. **(G-H)** Sequencing traces of PCR products of 2 False Negative calls (reference in 3 NGS and variant in ABI Sanger).
